# Religiosity/Spirituality and Mental Health in Older Adults: A Systematic Review and Meta-Analysis of Observational Studies

**DOI:** 10.3389/fmed.2022.877213

**Published:** 2022-05-12

**Authors:** Hélio José Coelho-Júnior, Riccardo Calvani, Francesco Panza, Riccardo F. Allegri, Anna Picca, Emanuele Marzetti, Vicente Paulo Alves

**Affiliations:** ^1^Università Cattolica del Sacro Cuore, Institute of Internal Medicine and Geriatrics, Rome, Italy; ^2^Department of Gerontology, Catholic University of Brasília, Brasília, Brazil; ^3^Fondazione Policlinico Universitario “Agostino Gemelli” IRCCS, Rome, Italy; ^4^National Institute of Gastroenterology “Saverio de Bellis”, Research Hospital, Bari, Italy; ^5^Department of Cognitive Neurology, Instituto de Investigaciones Neurológicas Fleni, Buenos Aires, Argentina; ^6^Department of Neurosciences, Universidad de la Costa (CUC), Barranquilla, Colombia

**Keywords:** religion, mental disorder (disease), depression, anxiety, elderly

## Abstract

**Objectives:**

The present study investigated the association between religious and spiritual (RS) practices with the prevalence, severity, and incidence of mental health problems in older adults.

**Methods:**

We conducted a systematic review and meta-analysis of cross-sectional and longitudinal studies that investigated older adults aged 60+ years and assessed RS using valid scales and questions from valid scales, and mental health according to validated multidimensional or specific instruments. Studies were retrieved from MEDLINE, LILACS, SCOPUS, CINAHL, and AgeLine databases until July 31, 2021. The risk of bias was evaluated using the Newcastle-Ottawa Quality Assessment Scale (NOS). A pooled effect size was calculated based on the log odds ratio (OR) and Z-scores. This study is registered on PROSPERO.

**Results:**

One hundred and two studies that investigated 79.918 community-dwellers, hospitalized, and institutionalized older adults were included. Results indicated that high RS was negatively associated with anxiety and depressive symptoms, while a positive association was observed with life satisfaction, meaning in life, social relations, and psychological well-being. Specifically, people with high spirituality, intrinsic religiosity, and religious affiliation had a lower prevalence of depressive symptoms. In relation to longitudinal analysis, most studies supported that high RS levels were associated with a lower incidence of depressive symptoms and fear of death, as well as better mental health status.

**Conclusion:**

Findings of the present study suggest that RS are significantly associated with mental health in older adults. People with high RS levels had a lower prevalence of anxiety and depressive symptoms, as well as reported greater life satisfaction and psychological well-being, better social relations, and more definite meaning in life. Data provided by an increasing number of longitudinal studies have supported most of these findings.

## Introduction

Mental health is an integral and vital component of health that encompasses emotional, psychological, and social wellbeing (1). It refers to the way by which people behave, identify their selves, and cope with stressful events (1, 2), thereby affecting how they experience and understand life events (1, 2). In contrast, mental health decline is accompanied by a high frequency of persistent negative emotions, thoughts, and beliefs that influence the quality of life (3).

Older adults are highly susceptible to mental health problems (4). It is expected that one in five seniors will experience some form of mental illness during late life (1). Depression, anxiety, and substance abuse are the most common conditions found in older adults (5–8), although many other syndromes, such as sleep disorders, apathy, and agitation/aggression have also been frequently observed (9). The evolution of mental disorders is disastrous to patients and commonly involves disability and suicide (10–12), in addition to its high economic costs (12).

This scenario was noted by the World Health Organization (WHO) (2, 3), which established the maintenance of mental health in older adults as a priority for healthcare professionals and societies around the world. However, this topic is a relatively recent issue in medical and biological sciences and is still poorly addressed (13), impeding the creation and establishment of specific evidence-based treatments that effectively embrace all patients' needs.

Religious and spiritual (RS) beliefs are far from being just cultural traditions. Indeed, it involves numerous organizational, non-organizational, introspective, and community practices that might potentially influence human behavior (14). Concerning mental health, an increasing number of studies have observed better mental health status in people involved in RS activities (15–35). These results have been further confirmed by descriptive and systematic reviews (36–45). However, a specific pooled analysis based on a systematic search focused on the old population and incorporating a variety of religious and spiritual approaches is still needed (46).

Such research has important health and policy implications, given that older adults are highly involved in RS activities (47), and have frequently mentioned that they would feel better if health professionals could understand and take into consideration their personal beliefs (48, 49).

Based on these premises, the present study investigated the association between RS and the prevalence, severity, and incidence of mental health-related conditions in older adults by conducting a systematic review and meta-analysis.

## Materials and Methods

This is a systematic review and meta-analysis of observational studies that investigated the cross-sectional and longitudinal associations between RS and mental health parameters in older adults. The study was fully performed by investigators and no librarian was part of the team. This study complies with the criteria of the Meta-analysis of Observational Studies in Epidemiology (MOOSE) guidelines (50) and the Cochrane Handbook for Systematic Reviews and Interventions (51). An a priori protocol was established and registered on PROSPERO, an international prospective register of systematic reviews [CRD42021292170].

### Eligibility Criteria

Case-control, cross-sectional and longitudinal cohort studies that examined as a primary or secondary outcome the association between RS and mental health parameters in people aged 60+ years were included. Additional eligibility criteria consist of: (a) using valid scales or subscales (facets), questions from valid scales, and self-report devotion, identification, and/or adherence for measuring religiosity and/or spirituality; (b) mental health parameters assessed using valid multidimensional (e.g., Short Form Health Survey [SF-36]) or specific (Center for Epidemiological Studies-Depression [CES-D]) instruments; (c) published studies in English, Italian, Spanish or Portuguese languages. Studies that investigated patients with depression were included if they contained a non-depressive control group or presented depression severity scores. To be included in the meta-analysis, investigations should provide sample size of at least two groups divided according to mental health and RS. Alternatively, studies were incorporated into the pooled analysis if Pearson's correlation (*r*), Z-scores, Beta (β), or Odds Ratio (OR) values, as well as their corresponding confidence intervals (CI) or standard error (SE), for the association between RS and mental health parameters were presented. Studies designed to review the literature, investigate psychometric properties, validate and/or translate instruments were excluded. We also excluded studies that compared the prevalence of mental health parameters among different religious affiliations, but not provided comparisons with no religious identification or atheism.

### Search Strategy and Selection Criteria

Studies published on or before July 31, 2021, were retrieved from the following five electronic databases by one investigator: MEDLINE (PubMed interface), LILACS (Virtual Health Library interface), Scopus (EBSCO interface), AgeLine (EBSCO interface), CINAHL (EBSCO interface). Further eligible articles were identified by checking reference lists of systematic reviews and meta-analyses (Supplementary Material 1) and retrieved articles. In addition, citation searches on key articles were performed in Google Scholar and ResearchGate. Initially, a search strategy was designed using keywords, MeSH terms, and free text words, such as Religion, Mental Health, and Older Adults. Additionally, keywords and subject headings were exhaustively combined using Boolean operators. The complete search strategy used on PubMed is shown in Supplementary Material 2.

### Data Extraction, Quality Assessment, and Risk of Bias

Titles and abstracts of retrieved articles were screened for eligibility by two researchers (HJCJ, RC). The full-text was consulted if the abstract did not provide enough information for final evaluation. Two reviewers (HJCJ, RC) extracted coded variables (i.e., methodological quality, risk of bias, and the characteristics of the studies) using a standardized coding form. A third researcher was consulted to solve disagreements (VP), if necessary. After extraction, data were divided into subcategories according to the type of religiosity and/or spiritual parameter and mental health aspects. To note, RS was examined as (a) Religious affiliation (yes/no), (b) Religious devotion (e.g., very much, not at all), (c) Religious index, (d) Organizational religious activities (ORA), (e) Non-organizational religious activities (NORA), and (f) Intrinsic religiosity (IR). ORA involved public religious practices, church/temple/cult/ritual attendance, and group-related religious activities (52); NORA included private activities, such as prayer, meditation, religious media, and scriptures studies (52); IR integrated personal commitment and/or motivation with the divine/deity (e.g., God). Spirituality was investigated alone and in combination with IR (52). Mental health parameters were divided into the following subcategories: (a) alcohol consumption, (b) anxiety, (c) death anxiety, (d) depressive symptoms, (e) hopelessness, (f) life satisfaction, (g) meaning in life, (h) psychological well-being, (i) self-esteem, (j) social relations, (k) suicidal ideation, and (l) tobacco use. A subcategory denominated general mental health was formed by studies that used composite scores that aggregate two or more mental health parameters or subscales of health survey questionnaires. The quality of reporting for each study was performed by two researchers (HJCJ, RC) using the Quality Assessment Tool for Observational Cohort and Cross-sectional of the National Institute of Health (53). This tool contains 14 questions that assess several aspects associated with the risk of bias, type I and type II errors, transparency, and confounding factors. Items 6, 7, and 13 do not refer to cross-sectional studies and were removed from the quality analysis. The maximum scores for cross-sectional and prospective studies were 8 and 14, respectively. The agreement rate for quality assessment between reviewers was 99%. The risk of bias was assessed using the Newcastle-Ottawa Quality Assessment Scale (NOS) (54, 55), according to Cochrane Collaboration Group' recommendation (56). NOS examines potential bin on selection, comparability, and outcome. The overall score ranges from 0 to 10 for cross-sectional studies, and from 0 to 9 for case-control and cohort studies. Scores ≤ 4 were identified as high risk of bias, scores 5–6 as moderate risk of bias, and scores ≥7 indicated a low risk of bias (57).

### Statistical Analysis

The meta-analysis was conducted using Revman 5.4.1 (Cochrane Collaboration, Copenhagen, Denmark) and STATA 13 (StataCorp, College Station, TX, USA). Effect sizes (ESs) were measured using OR, CI, Z-scores, and SE. When data were not made available by authors, they were calculated according to Cochrane guidelines (51). Specifically, SE was estimated according to the following formula: $\frac{\left(CI\;upperboundCI\;lowerbound\right)}{3.92}$. OR were obtained from β coefficients or by calculating the number of participants allocated into high and low SR groups, and subsequently log-transformed (base 10) to be analyzed. Due to the variability of sample characteristics, a random-effect model was used to calculate the pooled logOR. Pearson's correlations were converted into Z-scores by calculating$:\;0.\;5\;*\log\left(\frac{\left(1\;+\;r\right)\;}{\left(1\;-\;r\right)\;}\right)$. Pooled Z-scores were calculated using the generic inverse variance (IV) method. A sensitivity analysis was performed based on the stratification technique according to RS aspects, including religious affiliation, religious devotion, religious index, service attendance, ORA, NORA, IR, and spirituality. Single pairwise comparisons were created when multiple studies referred to the same database using the formulas proposed by the Cochrane group (51). Publication bias was measured when at least ten studies investigated the same outcome (51) by examining funnel plots and the Egger's test for funnel plot asymmetry.

## Results

### Literature Search

**Figure 1:**
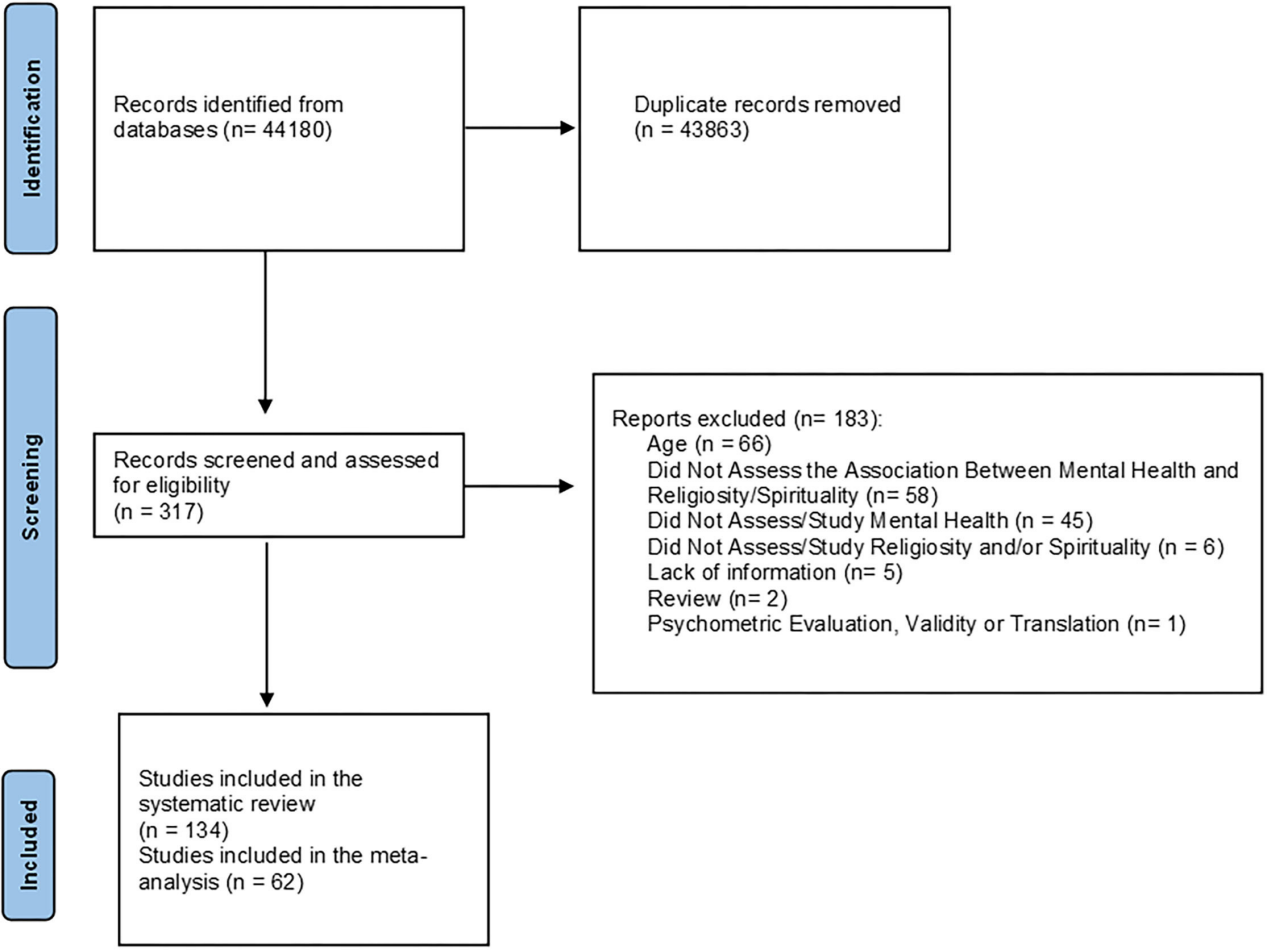
Flowchart of the present study.

Forty-four thousand one hundred and eighty records were identified through database and hand searching. Of these, 43,863 records were excluded based on duplicate data, title, and abstract. Three hundred and seventeen studies were fully assessed for eligibility and 183 articles were excluded (Supplementary Material 3). Finally, 134 studies were included in the qualitative analysis and 62 studies provided data to be included in the pooled analysis. The flowchart of the present study is shown in Figure 1.

### Cross-Sectional and Case-Control

A general description of the included cross-sectional and case-control studies that investigated religiosity is provided in Table 1. Religiosity was examined in one-hundred two studies (15–35, 46, 58–85, 87–90, 92–124, 126–136) that investigated 79,918 institutionalized, hospitalized, and community-dwellers with a mean age of ~73 years (from 60 to 90.3 years). Hospitalized patients included people with cancer, depression, and anxiety. Two studies involved older immigrants, one study investigated people who reported have experiencing trauma, and one study examined patients receiving home care nursing services. Investigations were conducted in Australia, Belgium, Brazil, Canada, Chile, China, Colombia, Egypt, Ghana, India, Iran, Italy, Japan, Korea, Lebanon, Malaysia, Mexican, Pakistan, Poland, Russia, Scotland, South Africa, South Korea, Spain, Sweden and Finland, Turkey, United Kingdom, and United States of America (USA) from 1977 to 2020. One study combined data from five different countries (i.e., England, Finland, Germany, Ireland, Netherland). The examined religious parameters included intrinsic and extrinsic religiosity, NORA, ORA, prayer, meditation, bible study, public religiosity, religious affiliation, religious behavior, religious coping, religious corporeity, religious devotion, religious doubt, religious engagement, religious index, religious involvement, religious knowledge, religious media, religious psychosocial resources, religious sentiment, religious support, service attendance, social religiosity, and private religious practices. Most studies (52.0%) assessed religiosity using self-developed scales, while 11.8% of the included studies used The Duke University Religion Index (DUREL), 5.9% The Religious Orientation Scale, 2.9% The Brief Multidimensional Measure Religiousness/Spirituality, 2.9% The Religious Coping Scale, 2% The Intrinsic Religiosity Scale, 2% The Religiosity index, and 2% The WHOQOL-SRPB. Other instruments, such as The Religious Attitudes Scale, The Religious Commitment Inventory, and The Religious Participation Questionnaire were only used in one study each. Mental health aspects included alcohol consumption, anger, anxiety, attachment to social relationships, cigarette smoking, coping strategies, death anxiety, depressive symptoms, emotional aspects limiting functioning, general psychological well-being, hopelessness, hostility, indirect life-threatening behavior, life satisfaction, meaning in life, optimism, satisfaction with life, self-esteem, social isolation, stress, suffering, and suicidal ideation. The majority of the mental health parameters were assessed using self-developed scales (26.5%) or specific instruments, including the GDS (21.6%), CES-D (12.7%), Hamilton Rating Scale for Depression (3.9%), Death Anxiety Scale (3.9%), Life Satisfaction Scale (2.9%), Beck Anxiety Inventory (2%), Suicidal Ideation Scale (2%), and Beck Hopelessness Scale (2%).

**Table 1:** Main characteristics of the cross-sectional and case-control studies that investigated the association between religious activities and mental health.

**References**	**Country**	**Sample characteristics**	**Sample Size**	**Mean Age**	**Religious parameter**	**Assessment tool**	**Mental health parameter**	**Assessment Tool**
Aslan et al. (15)	Turkey	Patients with cancer	125	71.5	(a) Positive Religious Coping; (b) Negative Religious Coping	Religious Coping Scale	Life Satisfaction	Life Satisfaction Scale
Foong et al. (58)	Malaysia	Chronically ill community-dwellers	1,790	69.2	(a) Intrinsic religiosity; (b) Expressing religiosity	Religious Orientation Scale	Life Satisfaction	The Satisfaction with life scale
Bae (16)	Korea	Community-dwelling older adults	6,471	60–99	Religiosity	Self-developed scale	Depressive Symptoms	CES-D
Abdel-Hady and El-Gilany (59)	Egypt	Community-dwelling older adults	663	67.3	Religious Commitment	Religious Commitment Inventory	Tobacco use	Tobacco smoking history
Mitchell et al. (27)	USA	Community-dwelling older adults	887	74.0	Religiosity	Self-developed scale	Hopelessness	Self-developed four-item hopelessness scale
Molina et al. (60)	Brazil	Community-dwelling older adults	613	—	(a) Spirituality, (b) Religiousness and (c) Personal Beliefs	WHOQOL-SRPB	(a) Psychological well-being; (b) Social Relations; (c) Death and dying	(a) WHOQOL-BREF, (b) WHOQOL-OLD
Sharif et al. (61)	Iran	Community-dwelling older adults	504	69.5	Religiosity	Self-developed scale	Attachment to social relationships: (a) close; (b) depend; (c) anxiety	Adult Attachment Scale Revised
Solaimanizadeh et al. (62)	Iran	—	180	74.0	(a) Spiritual health; (b) Religious coping	(a) Spiritual Health Questionnaire; (b) Religious Coping Questionnaire	Death Anxiety	Death Anxiety Questionnaire
Gallardo-Peralta and Sánchez-Moreno (63)	Chile	Community-dwelling older adults	777	60+	Religiousness/spirituality	Brief Multidimensional Measure of Religiousness/spirituality	Depressive Symptoms	15-item Geriatric Depression Scale
Silva et al. (64)	Brazil	Community-dwelling older adults	69	60+	Religious orientation	Self-developed Scale	Psychological Well-Being	WHOQOL-BREF
Bakhtiari et al. (65)	Iran	Community-dwelling older adults	316	67.9	(a) Intrinsic religiosity; (b) Extrinsic religiosity	Religious Orientation Scale	Death Anxiety	Death Anxiety Scale
Bakan et al. (66)	Turkey	Community-dwelling older adults	250	70.0	Religious orientation	Religious Orientation Scale	Death Anxiety	Death Anxiety Scale
Fernández-Niño et al. (67)	China, Ghana, India, Mexico, Russia and South Africa.	Community-dwelling older adults	1,281	69.9	Religious orientation	“Do you belong to a religious denomination?”	Depressive Symptoms	Self-developed algorithm
Hamid et al. (68)	Malaysia	Community-dwelling older adults who had experienced major life events	594	68.7	(a) Intrinsic religiosity; (b) Extrinsic religiosity	Religiosity Intrinsic-Extrinsic Scale	Depressive Symptoms	15-item Geriatric Depression Scale
Hill et al. (69)	Mexican	Community-dwelling older adults	2,479	73.0	Religious orientation	Self-developed scale	Social Support	Self-developed algorithm
Reyes-Ortiz et al. (70)	Colombia	—	19,004	69.2	Religiosity	Self-developed scale	Depressive Symptoms	15-item Geriatric Depression Scale
Strinnholm et al. (71)	Sweden and Finland	Community-dwelling older adults	1,014	90.3	Religious engagement	Self-developed scale	Depression	Experienced geriatrician
Willis et al. (72)	USA	Community-dwelling older adults	993	77.4	Religious Doubt	Self-developed scale	Death Anxiety and Depressive Symptoms	Self-developed scale
Moreno et al. (73)	Mexico	Community-dwelling older adults	39	71.0	Religiosity	DUREL	Alcohol consumption	Michigan Alcoholism Screening Test-Version Geriatric
Ejiri et al. (74)	Japan	Community-dwelling older adults	1,070	74.5	Religiosity	Self-developed scale	Social isolation	Self-developed scale
El-Gilany et al. (75)	Egypt	Older adults from rural and urban areas	474	67.3	Religious Commitment	RCI-10	Depressive Symptoms	15-item Geriatric Depression Scale
Kotian et al. (76)	Indian	—	9,836	60+	Religious orientation	(a) Hindu; (b) Muslim; (c) Christian; (d) others	Social Isolation	Self-developed scale
Manning and Miles (77)	USA	Noninstitutionalized older adults			(a) Religious Psychosocial Resources, (b) Religious Involvement	Self-developed scale	Depressive Symptoms	CESD
Nery et al. (78)	Brazil	Hospitalized older adults	140	60+	Religiosity	DUREL	Depressive Symptoms	15-item Geriatric Depression Scale
Munawar and Tariq (79)	Pakistan	Community-Dwelling older adults	100	68.3	Religiosity	The Religious Personality Scale of Muslim Religiosity-Personality Measurement Inventory	Satisfaction with Life	Satisfaction with Life Scale
Jung et al. (29)	South Korea	Community-dwelling older adults	622	76.6	Religiosity	DUREL	(a) Suicidal ideation; (b) Depressive symptoms	(a) Suicidal Ideation Scale; (b) Short Form Geriatric Depression Scale–Korean Version
Lac et al. (80)	Australia	Hospitalized older adults	100	83.0	Religiosity	Modified DUREL	Depressive Symptoms	15-item Geriatric Depression Scale
Nunes et al. (81)	Brazil	Community-dwelling older adults	100	80+	Spirituality, Religiousness and Personal Beliefs	WHOQOL-SRPB	(a) Depressive Symptoms; (b) Satisfaction With Life	(a) 15-item Geriatric Depression Scale; (b) Satisfaction With Life Scale
Bonnewyn et al. (82)	Belgium	Older inpatients	113	74.0	Religiosity	DUREL	Wish to die	Beck Scale for Suicide Ideation
McGowan et al. (83)	USA	Community-dwelling older adults	143	65+	(a) Organizational religiosity; (b) Intrinsic religiosity	(a) The Organizational Religiosity Scale; (b) The Intrinsic Religiosity Scale	(a) Depressive Symptoms; (b) Anxiety	Brief Symptom Inventory
Krok (84)	Poland	Community-dwelling older adults	212	71.04	Religiosity	Religious Meaning System Questionnaire	Coping strategies	Coping Inventory for Stressful Situations
Vieira and de Aquino (30)	Brazil	Community-dwelling older adults	100	67.1	(a) Religious knowledge; (b) Religious behavior; (c) Religious sentiment; (d) Religious corporeity	(a) Religious Attitudes Scale	Meaning in Life	The Meaning in Life Questionnaire
Abdala et al. (31)	Brazil	Community-dwelling older adults	911	71.8	Religiosity	(a) Organizational religiousness; (b) non-organizational religiousness; (c) intrinsic religiousness	Mental Health	Mental components of HRQOL
Fastame et al. (32)	Italy	Community-dwelling older adults	406	60–100	Religiosity	Religiosity index	General psychological well-being	The Psychological Well-Being and Aging Questionnaire
Stecz and Kocur (33)	Poland	Hospitalized older adults	61	70.9	(a) Religiosity; (b) Religious Practices	Mini-Cuestionario de Calidad Vida em Hipertensión Arterial	(a) Satisfaction with life; (b) Anxiety; (c) Stress	(a) The Satisfaction with Life Scale; (b) The State–Trait Anxiety Questionnaire; (c) The Perceived Stress Scale
Andrade et al. (85)	Brazil	Community-dwelling older adults	294	60+	Religious orientation	Self-developed scale	Mental Health	Mini-Cuestionario de Calidad Vida em Hipertensión Arterial
Feng et al. (86)	China	Older adults from rural and urban areas	1,329	60+	Religious belief	Yes/No	Depressive Symptoms	Geriatric Mental State Schedule
Chaves et al. (87)	Brazil	Community-dwelling older adults	287	72.0	Religiosity	DUREL	Depressive Symptoms	15-item Geriatric Depression Scale
dos Santos et al. (88)	Brazil	Community-dwelling older adults	82	71.0	Religiosity	DUREL	(a) Mental Health; (b) Emotional Aspects Limiting Functioning	SF-36
Hayward et al. (89)	USA	Older adults who reported experiencing trauma	206	66+	Religious Doubt	Self-developed scale	Depressive Symptoms	CES-D
Hayward and Krause (89)	USA	Community-dwelling older adults	1,011	79.1	Church Attendance	Self-developed scale	Hostility	Cook–Medley Hostility Scale
Lee and Lifen (34)	USA	Older Immigrants	246	76.2	Private Religious Practice	Brief Multidimensional Measures of Religiousness/Spirituality	(a) Mental Health; (b) Emotional Aspects Limiting Functioning	SF-36
Mefford et al. (35)	USA	Community-dwelling older adults	82	73.8	Religiosity	Brief Multidimensional Measure of Religiousness/Spirituality	Healthier anger management	Deffenbacher Anger Scale
Rivera-Ledesma et al. (90)	Mexico	Community-dwelling older adults	193	60–60.8	Spiritual Well-Being	Escala de Bienestar Espiritual	(a) Depressive Symptoms; (b) Anxiety; (c) Death Anxiety; (d) Suicide	(a) CES-D; (b) Inventario de Ansiedad de Beck; © Escala de Ansiedad ante la Muerte; (d) Escala de Ideación Suicida
Hafeez and Rafique (91)	Pakistan	Institutionalized older adults	60	60+	(a) Religiosity; (b) Spirituality	(a) Revised Religious Orientation Scale; (b) Spirituality Transcendence Scale	Psychological Well-Being	Self-developed scale
Ysseldyk et al. (92)	Canada	Institutionalized older adults	42	86.3	Religious identity	Self-developed scale	Depressive Symptoms	15-item Geriatric Depression Scale
Barricelli et al. (93)	Brazil	Community-dwelling older adults	60	73.0	(a) Intrinsic religiosity; (b) Extrinsic religiosity	Religious Orientation Scale	(a) Mental Health; (b) Emotional Aspects Limiting Functioning	SF-36
Jahn et al. (94)	USA	Community-dwelling older adults	272	72.8	Religiosity	DUREL	(a) Depressive Symptoms; (b) Suicide	(a) CES-D; (b) Geriatric Suicide Ideation Scale.
Krause and Bastida (95)	USA	Community-dwelling older adults	509	73.4	Church Attendance	Self-developed scale	Personal Control	Self-developed scale
Momtaz et al. (96)	Malaysia	Community-dwelling older adults	1,415	60.0	(a) Intrinsic religiosity; (b) Extrinsic religiosity	Religious Orientation Scale	Mental Health	WHO-5 Well-Being Index
Moon and Kim (97)	South Korea	Community-dwelling older adults	274	76.7	Religiosity	DUREL	(a) Depressive Symptoms	Short Geriatric Depression Scale-Korean version
Richardson et al. (98)	USA	Community-dwelling older adults	378	76.5	Religiosity	Self-developed scale	Depression	Diagnostic and Statistical Manual of Mental Disorders
Vitorino and Vianna (99)	Brazil	Institutionalized older adults	77	76.6	Religiosity	Religious/Spiritual Coping Scale	Coping strategies	Religious/Spiritual Coping Scale
Park et al. (100)	USA	Community-dwelling older adults	207	72.5	Religiosity	The Brief Multidimensional Measure of Religiousness/Spirituality	Life Satisfaction	The Satisfaction with Life Scale
Callen et al. (101)	USA	Community-dwelling older adults	82	74.0	Religiosity	The Brief Multidimensional Measure of Religiousness/Spirituality	Stress	Perceived Stress Scale
Corrêa et al. (102)	Brazil	Community-dwelling older adults	1,980	71.9	Church Attendance	Self-developed scale	Common Mental Disorders	Psychiatric Screening Questionnaire-20
Krause and Bastida (103)	USA	Community-dwelling older adults	1,005	66+	Religiosity	Self-developed scale	(a) Suffering; (b) Optimism	Self-developed scale
Lucchetti et al. (104)	Brazil	Older patients	484	68.9	Religiosity	Private and Social Religious Practice Scale	(a) Depressive Symptoms; (b) Anxiety	(a) 15-item Geriatric Depression Scale; (b) Diagnostic and Statistical Manual of Mental Disorders
Schieman et al. (105)	USA	Community-dwelling older adults	1,167	65+	Religiosity	Self-developed scale	Sense of Mattering	Self-developed scale
Idler et al. (106)	USA	—			(a) Religious attendance; (b) Deeply religious	Self-developed scale	Depressive Symptoms	CESD
McFarland et al. (107)	USA	Community-dwelling older adults	919	74.4	Religiosity	Self-developed scale	(a) Depressive Symptoms; (b) Death anxiety; (c) Optimism; (d) Self-esteem	Self-developed scale
Cardoso and Ferreira (17)	Brazil	Community-dwelling older adults	256	69.1	Religiosity	Chatters scale	Life Satisfaction	PANAS scale
Scandrett and Mitchell (18)	USA	Institutionalized older adults	140	85.4	(a) Positive Religious Coping; (b) Negative Religious Coping	Self-developed scale	Psychological Well-Being	Bradburn Affect Balance Scale
Cruz et al. (19)	USA	Older adults with depression	130	71.9	(a) Frequency of Church attendance; (b) Frequency of prayer	Self-developed scale	(a) Depression severity; (b) hopelessness	Hamilton Rating Scale for Depression-17; Beck Hopelessness Scale
Bishop (20)	USA	Older Residents in Religious Monasteries	235	78.3	Attachment to God	The Attachment to God Scale	Depressive Symptoms	10-item Geriatric Depression Scale
Blay et al. (108)	Brazil	Community-dwelling older adults	6,961	60+	(a) Religious orientation; (b) Social religiosity	Self-developed scale	a) Depressive Symptoms; b) Current tobacco use; c) Alcohol abuse or dependence	(a) Short Psychiatric Evaluation Schedule; (b) “Do you use tobacco regularly now?”; (c) Self-Reported Scale
Hara et al. (21)	Brazil	Community-dwelling older adults	1,541	69.9	Religious orientation	Self-developed scale	Excessive daytime sleepiness	Self-developed scale
Payman et al. (109)	Australia	Community-dwelling older adults	86	76.6	Religiosity	DUREL	Depressive Symptoms	15-item Geriatric Depression Scale
Reyes-Ortiz et al. (22)	USA	Community-dwelling older adults	3,050	72.7	Church attendance	Self-developed scale	Depressive Symptoms	15-item Geriatric Depression Scale
Chaaya et al. (110)	Lebanon	Community-dwelling older adults	740	60+	Religiosity	Self-developed scale	Depressive Symptoms	15-item Geriatric Depression Scale
Dunn (111)	USA	Community-dwelling older adults	92	74.0	Religiosity	Self-developed scale	Depressive Symptoms	15-item Geriatric Depression Scale
King et al. (112)	USA	Community-dwelling older adults	675	75.2	Religiosity	DUREL	Depressive Symptoms	24-item Hamilton Rating Scale for Depression
Keyes and Reitzes (113)	USA	Community-dwelling older adults	242	65.2	(a) Religious attendance; (b) Religiosity	Self-developed scale	(a) Depressive Symptoms; (b) Self-esteem	(a) 15-item Geriatric Depression Scale; (b) 10-item self-esteem scale
Yoon and Lee (114)	USA	Older adults from rural areas	215	65+	(a) Spiritual experiences; (b) Private religious practice; (c) Religious and spiritual coping; (d) Religious support	Brief Multidimensional Measures of Religiousness/Spirituality	(a) Depressive Symptoms; (b) Satisfaction with Life	(a) Center for Epidemiological Studies-Depression (CES-D); (b) SWLS
Chen et al. (115)	USA	Older adults with depression and/or anxiety	1,610	74	Frequency of participation in religious activities	Self-developed scale	(a) Depressive Symptoms; (b) Anxiety	(a) CES-D; (b) Beck Anxiety Inventory
Mui and Kang (116)	USA	Older Immigrants	407	65+	Religiosity	Self-developed scale	Depressive Symptoms	Self-developed scale
Roff et al. (117)	USA	Community-dwelling older adults	973	65–106	Religiosity	DUREL	Cigarette Smoking	Self-developed scale
Bosworth et al. (118)	USA	Older adults with depression	114	67.4	(a) Public Religious Practice; (b) Private Religious Practice; (c) Positive Religious Coping; (d) Negative Religious Coping	Self-developed scale	Depression severity	Montgomery Asberg Depression Rating Scale
Meisenhelder (119)	USA	Community-dwelling older adults	271	74.4	(a) Frequency of prayer	Self-developed scale	(a) Mental Health; (b) Emotional Aspects Limiting Functioning	SF-36
Milstein et al. (120)	USA	Patients receiving home care nursing services	130	78.7	Religiousness/Spirituality	Self-developed scale	Depression	DSM-IV
Parker et al. (46)	USA	Community-dwelling older adults	1,000	65–106	Religiosity	DUREL	(a) Depressive Symptoms; (b) Mental Health	(a) 15-item Geriatric Depression Scale; (b) SF 12
Blazer et al. (121)	USA	Older adults from rural areas	1,155	73.1	(a) Public religion; (b) Religious media use; (c) Private religion	Self-developed scale	Alcohol consumption	Self-developed scale
Cicirelli (122)	USA	Community-dwelling older adults	388	72.6	Religiosity	Self-developed scale	(a) Fear of Death; (b) Self-esteem	(a) Multidimensional Fear of Death Scale; (b) Self-esteem
Herrera et al. (123)	Spain	Institutionalized older adults	41	77.4	Religiosity	Coping Factors Scale	(a) Mental Health; (b) Depressive Symptoms	(a) Self-developed scale; (b) 30-item Geriatric Depression Scale;
Braam et al. (124)	Europe	Community-dwelling older adults	8,398	74.6	(a) Church attendance; (b) Religious orientation	Self-developed scale	Depressive Symptoms	Geriatric Mental State Examination, short a version of the Comprehensive Assessment and Referral Evaluation, CES-D, DSM-IV
Fry (23)	Canada	Community-dwelling older adults	188	65–89	(a) Religiosity; (b) Spirituality	a) Self-developed scale; b) Index of Spirituality	Psychological Well-Being	Self-developed scale
Musick et al. (125)	USA	Community-dwelling older adults	1,897	65+	Religiosity	Self-developed scale	(a) Depressive symptoms; (b) Alcohol use	(a) CESD; (b) Self-developed scale
Guglani et al. (126)	United Kingdom	Community-dwelling older adults	—	66.9	Religious Participation	Religious Participation Questionnaire	(a) Anxiety; (b) Depression;(c) Self-Esteem	(a) and (b) Hospital Anxiety and Depression Scale; (c) Rosenberg Self-Esteem Scale
Menon et al. (127)	USA	Hospitalized older adults	295	70.5	Religious orientation	Self-developed scale	(a) Depression; (b) Hopelessness; Life Satisfaction	a) Geriatric Depression Scale,20 the Hamilton Rating Scale for Depression, and the DSM-III-R; (b) Beck Hopelessness Scale; (c) Life Satisfaction Scale
Husaini et al. (128)	USA	Community-dwelling older adults	995	72.0	(a) Public Religiosity; (b) Private Religiosity	Self-developed scale	Depressive Symptoms	CES-D
Koenig et al. (129)	USA	Community-dwelling older adults	3,968	73	(a) Attendance at Religious Services; (b) Private Prayer, Meditation, Bible study; (c) Religious media	Self-developed scale	Cigarette Smoking	Self-developed scale
Musick et al. (130)	USA	Older adults from rural and urban areas	3,007	64–100	(a) Religious media; (b) Religious devotion	Self-developed scale	Depressive Symptoms	CES-D
Tapanya et al. (131)	Canada	Community-dwelling older adults	104	71.0	(a) Intrinsic religiosity; (b) Extrinsic religiosity	Age Universal I-E Scale	Worry	Penn State Worry Questionnaire
Kennedy et al. (132)	USA	Community-dwelling older adults	1,855	65+	Religious orientation	Self-developed scale	Depressive Symptoms	CES-D
Krause (133)	USA	Community-dwelling older adults	1,005	74.1	(a) Organizational religiosity; (b) Non-organizational Religiosity; (c) Religious Coping	Self-developed scale	Self-Esteem	Self-developed scale
Koenig et al. (134)	USA	Hospitalized older adults	850	69.8	(a) Religious coping index score; (b) Religious orientation	Self-developed scale	(a) Alcohol use; (b) Depressive Symptoms	(a) Self-developed scale; (b) GDS and Hamilton depression scale
Pressman et al. (135)	USA	Hospitalized older adults	31	65+	Religiosity	Index of Religiousness	Depressive Symptoms	15-item Geriatric Depression Scale
Thorson and Powell (24)	USA	Community-dwelling older adults	103	70.8	Religiosity	10-item Intrinsic Religious Motivation scale	Death anxiety	Death anxiety scale
Guy (25)	USA	Community-dwelling older adults	1,170	60+	Church Attendance	Self-developed scale	Life Satisfaction	Life Satisfaction Index A
Hunsberger (136)	Canada	Community-dwelling older adults	75	65–88	Church attendance	Self-developed scale	Life Satisfaction	Self-developed scale
Nelson (26)	USA	Institutionalized older adults	58	66.2	(a) Religious commitment; (b) Religious orientation	Self-developed scale	Indirect life-threatening behavior	Self-developed scale
Reid et al. (28)	Scotland	Community-dwelling older adults	501	65+	Religious orientation	Self-developed scale	Fear of death	Self-developed scale

*CESD, Center for Epidemiological Studies Depression; DSM, Diagnostic and Statistical Manual of Mental Disorders; DUREL, The Duke University Religion Index; HRQOL, Health-Related Quality of Life; RCI-10, The Religious Commitment Inventory-10; SF-36, Short Form Health Survey; SWLS, Satisfaction with Life Scale; USA, United States of America; WHOQOL-SRBP, World Health Organization Quality of Life Spirituality, Religiousness and Personal Beliefs*.

The main characteristic of the studies that investigated the association between spirituality and mental health is shown in Table 2. Twenty-seven studies investigated the association between spirituality and mental health parameters (63, 73, 100, 137–147, 149–161). Two investigations studied both religiosity and spirituality (63, 73). A total of 13,015 community-dwelling and institutionalized older adults with a mean age of 75.1 years (ranging from 70.3 to 83.1 years) from Belgium, Brazil, Bulgaria, Chile, England, Iran, Korea, Mexico, Pakistan, Portugal, Romani, Spain, Taiwan, Turkey, and the USA were studied. Native people and immigrants were investigated in one study each. Spirituality was assessed in 37.0% of the studies, spiritual well-being in 29.6%, RS in 26%, and spiritual belief in 7.4%. Spirituality was predominantly assessed using The Spiritual Well-being Scale (25.9%), followed by self-developed scales (18.5%), The Brief Multidimensional Measure of RS (18.5%), and The Spirituality Index of Well-Being (7.4%). Other scales were used in one study each. The studied mental health aspects included alcohol abuse, depressive symptoms, emotional aspects limiting functioning, happiness, hope, global mental health, optimism, fear of death and dying, positive mental well-being, psychological well-being, meaning in life, satisfaction with life, sense of suffering, and spiritual distress high heterogeneity was observed among the instruments used to assess mental health parameters. The most predominant scales were The CES-D, The GDS, The SF-36, seethe lf-developed scales, The Satisfaction with life scale, and The WHOQOL-BREF/WHOQOL-OLD.

**Table 2:** Main characteristics of the cross-sectional studies that investigated the association between spiritual activities and mental health.

**References**	**Country**	**Sample characteristics**	**Sample Size**	**Mean Age**	**Religious parameter**	**Assessment tool**	**Mental health parameter**	**Assessment Tool**
Khodarahimi et al. (137)	Iran	Community-Dwellers and Nursing home residents	120	64–93	Spirituality	Spiritual Well-Being Inventory	(a) Cognitive tolerance; (b) Happiness	(a) Multiple Stimulus Types Ambiguity Tolerance Scale-II; (b) Oxford Happiness Questionnaire
Aydin et al. (138)	Turkey	Community-Dwellers and Nursing home residents	144	60+	Spiritual Well-being	Spiritual Well-being Scale	Psychological well-being; (b) Meaning in Life Questionnaire	Symptom Checklist-90-R; (b) Meaning in Life
Fernandes et al. (139)	Portugal	Community-dwelling older adults	102	77.3	Spiritual Well-being	Spiritual Well-being Scale	Satisfaction With Life	Satisfaction With Life Scale
Gallardo-Peralta and Sánchez-Moreno (63)	Chile	Community-dwelling older adults	170	60+	religiousness/spirituality	Brief Multidimensional Measure of Religiousness/spirituality	Depressive Symptoms	GDS-30
Ilyas et al. (140)	Pakistan	Community-dwelling older adults	410	61+	Spirituality	Self-Developed Scale	Self Esteem	Self-Developed Scale
Hassoun et al. (141)	Spain	Older religious residents	435	83.1	Spiritual Well-being	Spiritual Well-being Scale	Sense of suffering	Escala Humanizar
Moreno et al. (73)	Mexico	Community-dwelling older adults	39	71.0	Spirituality; Religiosity	a Escala de Perspectiva Espiritual; DUREL	Alcohol abuse	Michigan Alcoholism Screening Test-Version Geriatric
Salman and Lee (142)	Taiwan	Community-dwelling older adults	150	65+	Spiritual well-being; (b)	Spirituality Index of Well-Being; (b) Spiritual Practices Checklist	Depressive Symptoms	CES-D
Thauvoye et al. (143)	Belgium	Community-dwelling older adults	279	76.0	Spirituality	Self-Developed Scale	Positive Mental Well-Being	Warwick-Edinburgh Mental Well-being Scale
Araújo et al. (144)	Portugal	Non-demented	80	101.0	Spirituality	Valuation of Life Scale	Satisfaction With Life	Satisfaction With Life Scale
Garces et al. (145)	Brazil	Community-dwelling older adults	241	60+	Spiritual Well-being	Spiritual Well-being Scale	Resilience	Resilience Scale
Lee and Salman (146)	Taiwan	Community-dwelling older adults	150	65+	Spiritual well-being	Spirituality Index of Well-Being	Depressive Symptoms	CES-D
Pilger et al. (147)	Brazil	Community-dwelling older adults	114	60–79	Spirituality	Spiritual Well-Being Scale	Psychological Well-Being; (b) Death and dying	WHOQOL-BREF; WHOQOL-OLD
Souza et al. (148)	Brazil	Community-dwelling older adults	301	60+	Spirituality; Religiosity	Escala de Espiritualidade de Pinto e Pais-Ribeiro; Self-Developed Scale	Hope	Hearth Hope Index
Vitorino et al. (149)	Brazil	Institutionalized Older Adults	148	76.6	religiousness/spirituality	spiritual/religious coping scale	Psychological Well-Being; (b) Death and dying	WHOQOL-BREF; WHOQOL-OLD
Ali et al. (150)	Iran	Community-dwelling older adults	141	60+	Spiritual Well-being	Spiritual Well-being Scale	Mental health	SF-36
Jun et al. (151)	USA	Older adults in assisted living facilities	224	65–102	Spirituality	Brief Multidimensional Measures of Religiousness/Spirituality	Depressive Symptoms	Geriatric Depression Scale–Short Form
Oliver et al. (152)	Spain	Community-dwelling older adults	224	75+	Spirituality	Functional Assessment of Chronic Illness Therapy	Satisfaction With Life; Hope;	satisfaction with life scale; Dispositional Hope Scale
Caldeira et al. (153)	Portugal	Older adults with cancer	45	70.3	Spiritual Well-being	Spiritual Well-being Scale	Spiritual distress	Self-Developed Scale
Park et al. (100)	USA	Community-dwelling older adults	200	72.5	religiousness/spirituality	Brief Multidimensional Measure of Religiousness/Spirituality	Depressive Symptoms	GDS-30
Coleman et al. (154)	Bulgaria and Romani	Older adults from Rural Areas	160	71.9	Spiritual belief	The Royal Free Interview for Religious and Spiritual Beliefs	Depressive Symptoms	Depression subscale of the Hospital Anxiety and Depression Scale
Lee and Yoon (155)	USA	Korean Immigrants	206	76.3	religiousness/spirituality	Brief Multidimensional Measures of Religiousness/Spirituality	Psychological Well-Being	General Well-Being Schedule
Vahia et al. (156)	USA	Community-dwelling older adults	1,942	73	Spirituality	Brief Multidimensional Measure of Religiosity/Spirituality	Mental Health; Resilience; Optimism; Depression	SF-36; Connor-Davidson Scale for Resilience; Life Orientation Test; CESD
Skarupski et al. (157)	USA	Community-dwelling older adults	6,534	74.2	religiousness/spirituality	Daily Spiritual Experiences	Depressive Symptoms	CES-D
You et al. (158)	Korea	Community-dwelling older adults	152	65–94	Spirituality	Self-Developed Scale; The Spirituality Index of Well-Being; Daily Spiritual Experiences Scale	Depression subscale of the Hospital Anxiety and Depression Scale	Profile of Mood Status
Kirby et al. (159)	England	Institutionalized Older Adults	233	80.0	Spiritual beliefs	Royal Free Interview for Spiritual and Religious Beliefs	Psychological Well-Being	Psychological Well-Being Scale
Meisenhelder and Chandler (160)	USA	Older Native Americans	71	75.0	Spirituality	Self-Developed Scale	(a) Mental Health; (b) Emotional Aspects Limiting Functioning	SF-36

*CESD, Center for Epidemiological Studies Depression; GDS, Geriatric Depression Scale; SF-36, Short Form Health Survey; USA, United States of America; WHOQOL, World Health Organization Quality of Life*.

Table 3 provides the main characteristics of the included longitudinal studies. One study provided cross-sectional and longitudinal data (92). Eight studies (92, 162–168) that involved 12,067 community-dwelling older adults with a mean age of 72.3 years (ranging from 68.3 to 75.6 years) from Australia, England, Korea, Taiwan, and the USA were included. RS parameters included attachment to God, religious participation, religious identity, religious orientation, religious activities, religious coping, and church attendance. These parameters were assessed using self-developed scales and the DURAL index. The main outcome studied was depressive symptoms (75%), followed by death anxiety (12.5%) and mental health (12.5%). Mental health parameters were assessed using self-developed scales, CES-D, GDS, and the National Institute of Mental Health (NIMH) Diagnostic Interview Schedule.

**Table 3:** Main characteristics of the longitudinal studies that investigated the association between religious/spiritual activities and mental health.

**References**	**Time of follow-up (years)**	**Study design**	**Country**	**Sample**	**Sample Size**	**Mean Age**	**Religious parameter**	**Assessment tool**	**Mental health parameter**	**Assessment Tool**
Jung (162)	3	Longitudinal	USA	Community-dwelling older adults	936	65+	Secure attachment to God	Self-developed scale	Death anxiety	Self-developed scale
Roh et al. (163)	3	Longitudinal	Korea	Community-dwelling older adults	6,647	69.8	Religiosity	Self-developed scale	Depressive Symptoms	GDS-15
Hui Chuan (164)	5	Longitudinal	Taiwan	Community-dwelling older adults	3,537	60+	(a) Religious orientation; (b) Religious activities; (c) Religious coping	Self-developed scales	Depressive Symptoms	CES-D
Ysseldyk et al. (92)	7	Longitudinal	England	Community-dwelling older adults	7,021	70.9	Religious identity	Self-developed scale	Mental Health	Self-developed scale
Sun et al. (165)	4	Longitudinal	USA	Community-dwelling older adults	1,000	75.0	Religiosity	DUREL	Depressive Symptoms	GDS
Law and Sbarra (166)	8	Longitudinal	Australia	Community-dwelling older adults	791	75.6	Church attendance	Self-developed scale	Depressive Symptoms	CESD
Norton et al. (167)	3	Longitudinal	USA	Community-dwelling older adults	2,989	73.8	Church Attendance	Self-developed scale	Depressive Symptoms	NIMH Diagnostic Interview Schedule
Kivelä et al. (168)	5	Longitudinal	Finland	Community-dwelling older adults	679	68.3	Religious Participation	Self-developed scale	Depressive Symptoms	Self-Developed scale

*CESD, Center for Epidemiological Studies Depression; GDS, Geriatric Depression Scale; NIMH, National Institute of Mental Health; USA, United States of America*.

### Quality Assessment and Risk of Bias

The overall quality assessment of cross-sectional studies is shown in Supplementary Material 4. Overall scores varied from 3 to 9. All studies defined the research question (item 1), clearly specified and defined the study population (item 2), and used valid and reliable instruments to assess RS (item 9) and mental health aspects (item 11). Most studies (58.7%) uniformly applied the eligibility criteria and recruited participants in the same population at the same period (item 4). On the other hand, different levels of exposition (35.7%; item 8) and sample size justification (15.1%; item 5) were only provided in some studies. Similarly, just 32.5% of the studies had a participation rate of at least 50% (item 3), while 30.2% adjusted results according to potential confounding variables (item 14), and only 1.6% of the investigations were conducted with assessors blinded to the exposure of participants (item 12). No studies assessed RS more than once (item 10). One study had a case-control design and received an overall quality score of 9 points.

The overall quality assessment of longitudinal studies is shown in Supplementary Material 5. Quality scores varied from 7 to 11. All studies defined the research question (item 1), clearly specified and defined the study population (item 2), measured the exposure of interest before the outcome being measured (item 6), used a sufficient timeframe to reasonably expect the development of the studied outcomes (item 7), and used valid and reliable instruments to assess RS (item 9) and mental health aspects (item 11). Most studies had a participation rate of at least 50% (63%; item 3), uniformly applied the eligibility criteria and recruited participants in the same population at the same period (88%; item 4), and measured different levels of exposition (63%; item 8). Results were adjusted according to potential confounding variables in 50% of the investigations (item 14). One study presented a loss to follow-up after baseline ≤ 20% (item 13). Neither of the studies justified the sample size (item 5) or assessed RS more than once (item 10). Any investigation described if assessors were blinded to exposure (item 12).

The detailed risk of bias assessment is shown in Supplementary Material 6. The overall risk of bias in cross-sectional studies ranged from 3 to 9 (low to high). As per protocol, all studies used validated or well-described tools to measure RS and mental health aspects. Insufficient sample size justification was the most prevalent bias (84.1%), followed by unsatisfactory statistical analysis (69.0%), insufficient description of respondents' rate (66.7%), and representativeness of the studied sample (42.1%). The case-control study had a mean risk of bias classified as moderate.

Cohort studies had a mean risk of bias that ranged from 5 to 8 points (good). All studies investigated sample representatives or somewhat representatives of the population, recruited participants of the non-exposed cohort from the same community, used a structured interview for assessing the exposure, demonstrated that the outcome of interest was not present at the start of the study, and used a follow-up long enough to allow the occurrence of study outcomes. However, only 25% of the investigations assessed study outcomes using an independent blind assessor, while most studies used self-report. Finally, most of the studies (87.5%) had a follow-up rate of <80% or did not provide a clear statement.

### Cross-Sectional Associations Between RS and Mental Health Aspects in Older Adults

#### Alcohol Consumption

Five studies investigated the association between RS and alcohol consumption (73, 108, 121, 125, 134). Moreno et al. (73) observed that religiosity, but not spirituality, was negatively associated with alcohol consumption in Mexican older adults. In addition, Koenig et al. (134) observed that religious coping was negatively associated with alcohol abuse in old males. Musick et al. (125) noted that Baptists' older adults who lived in rural areas were less likely to drink alcohol than their counterparts from urban areas. According to the authors, these results occurred in function of higher service attendance levels. Analyzing the same cohort, Blazer et al. (121) expanded previous results by indicating that public religious activities were associated with alcohol abstinence in white and black Baptists. In contrast, Blay et al. (108) did not observe significant associations between religious affiliation, and social religiosity, have experienced religious changes, and have religion as an orienting-motivating force with alcohol abuse and dependency in Brazilian older adults.

#### Anxiety

**Figure 2:**
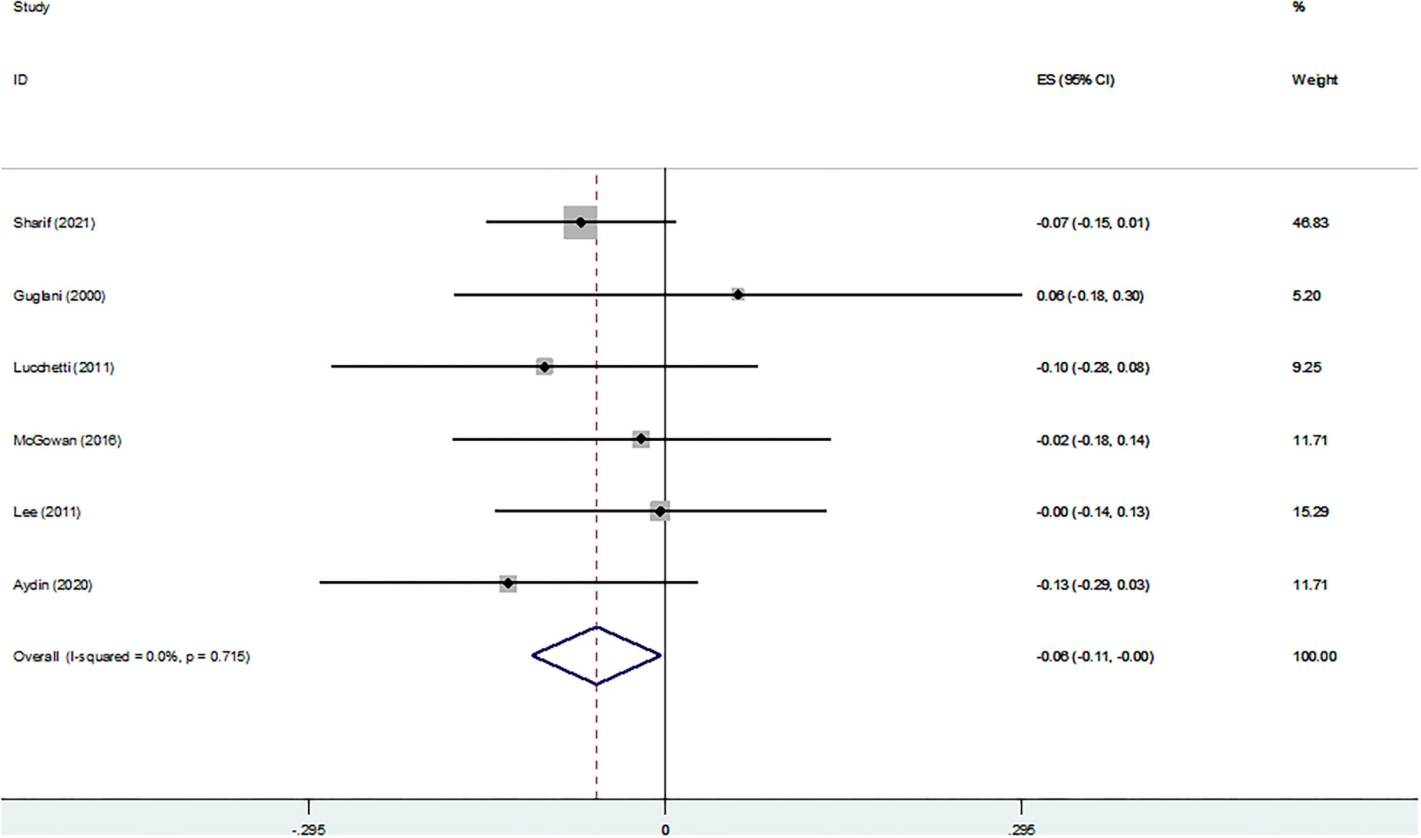
Z-scores for anxiety.

The association between RS and anxiety is shown in Figure 2. The pooled analysis of six studies indicated a significant and negative association between RS and anxiety (Z-score = −0.057, 95 % CI = −0.111–0.003, *P* = 0.037).

#### Death Anxiety

**Figure 3:**
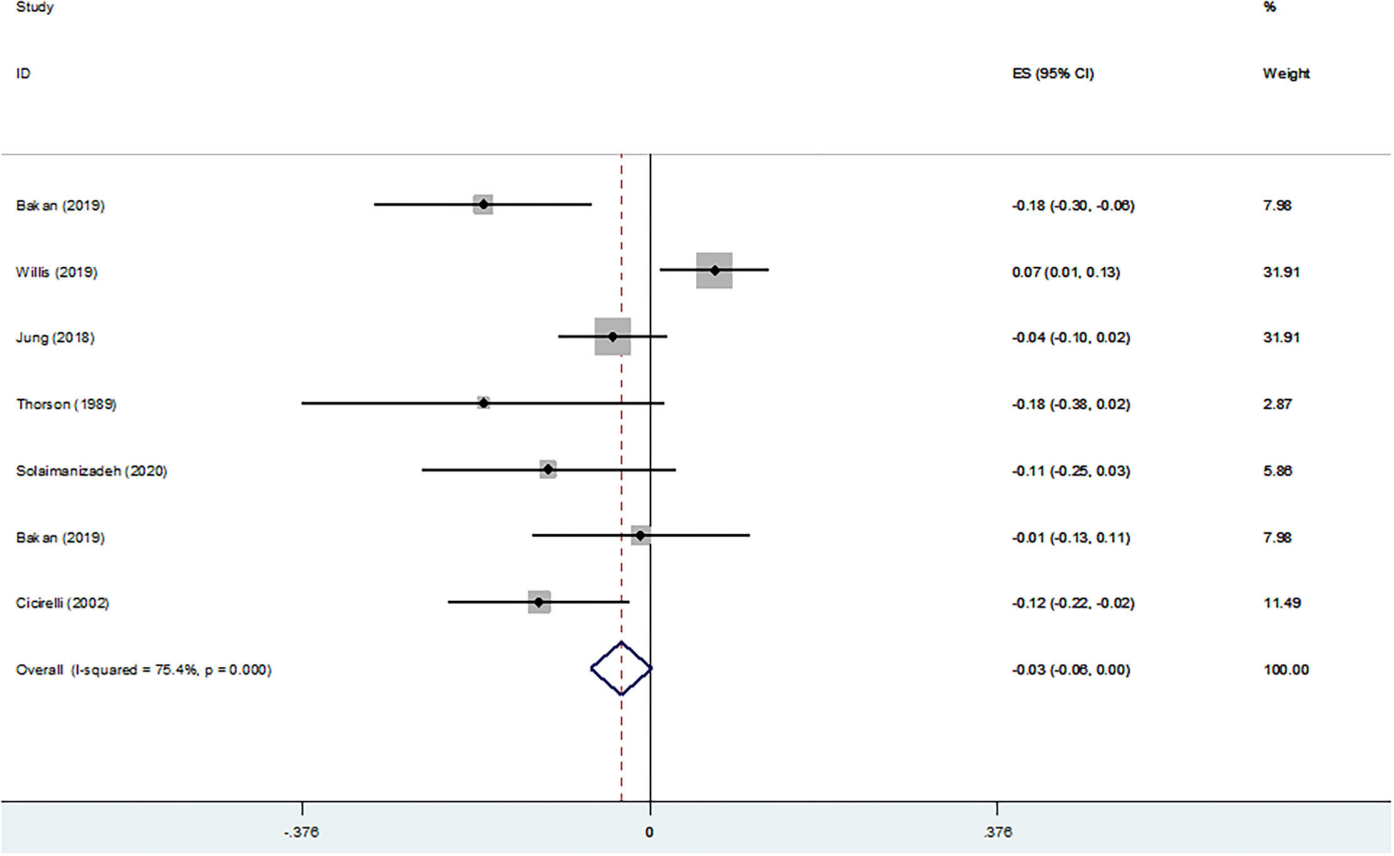
Z-scores for death anxiety.

Figure 3 shows the association between religiosity and death anxiety. The pooled analysis of seven studies indicated a non-significant association (Z-score = −0.031, 95 % CI = −0.064–0.002, *P* = 0.067).

#### Depressive Symptoms

**Figure 4:**
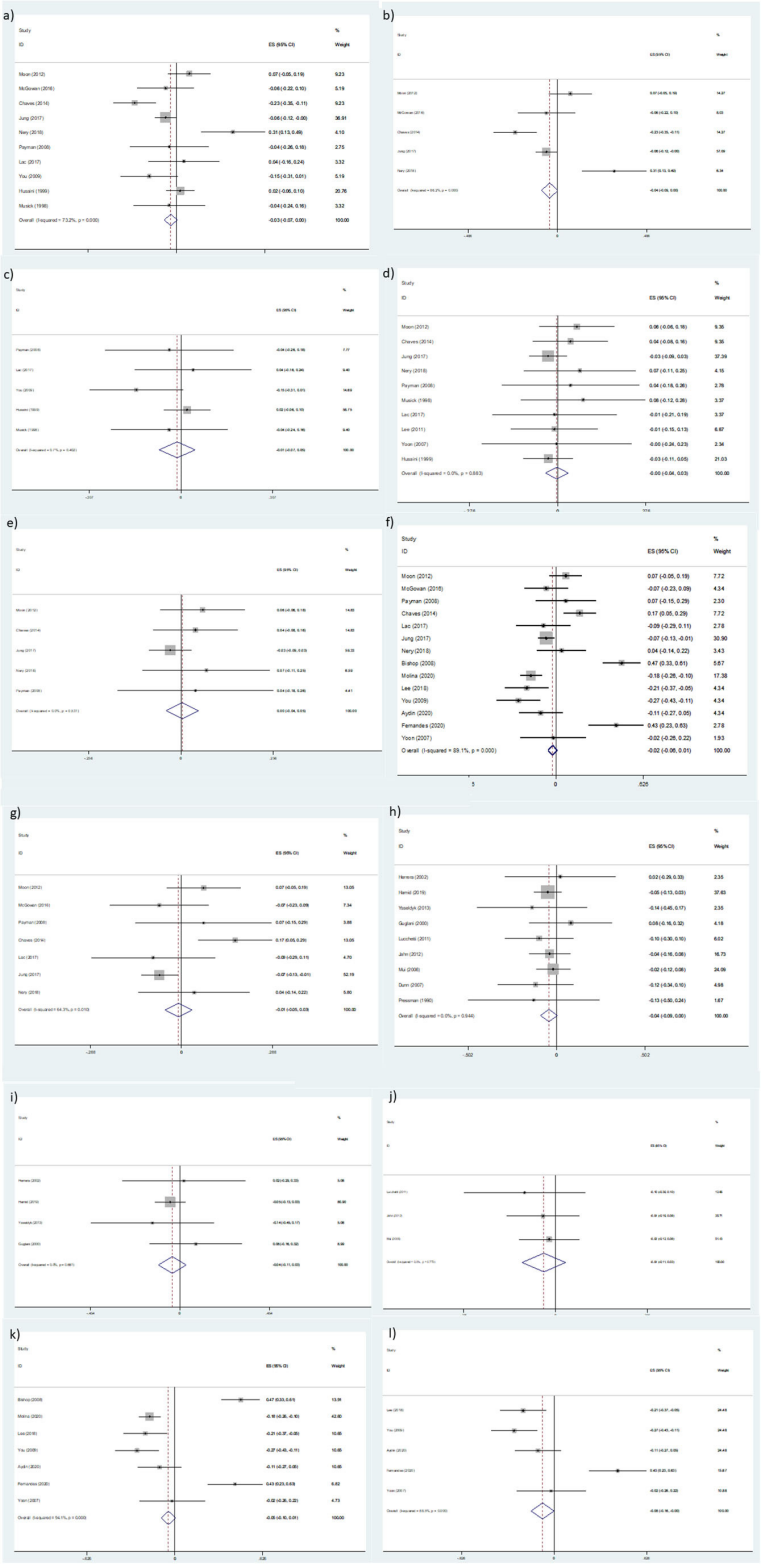
Z-scores for **(A)** ORA, **(B)** ORA-based DUREL scores, **(C)** service attendance, **(D)** NORA, **(E)** NORA-based DUREL scores, **(F)** IR, **(G)** IR-based DUREL scores, **(H)** religious, **(I)** religious, **(J)** religious devotion, **(K)** spiritual aspects, and **(L)** spirituality.

Figure 4 shows the cross-sectional association between RS and depressive symptoms according to continuous data. A non-significant association between ORA (Z-score = −0.032, 95 % CI = −0.068–0.004, *P* = 0.079), ORA-based DUREL scores (Z-score = −0.042, 95 % CI = −0.087–0.002, *P* = 0.062), service attendance (Z-score = −0.013, 95 % CI = −0.073–0.047, *P* = 0.662), NORA (Z-score = −0.003, 95 % CI = −0.039–0.033, *P* = 0.889), NORA-based DUREL scores (Z-score = 0.003, 95 % CI = −0.042–0.049, *P* = 0.883), IR (Z-score = −0.024, 95 % CI = −0.057–0.008, *P* = 0.145), IR-based DUREL scores (Z-score = −0.010, 95 % CI = −0.052–0.033, *P* = 0.660), religious (Z-score = −0.044, 95 % CI = −0.092–0.004, *P* = 0.073), religious affiliation (Z-score = −0.039, 95 % CI = −0.110–0.031, *P* = 0.274), religious devotion (Z-score = −0.037, 95 % CI = −0.108–0.033, *P* = 0.297), and spiritual aspects (Z-score = −0.046, 95 % CI = −0.097–0.005, *P* = 0.080) and depressive symptoms was observed. In contrast, a significant and negative association was observed with spirituality (Z-score = −0.079, 95 % CI = −0.157–0.002, *P* = 0.045).

**Figure 5:**
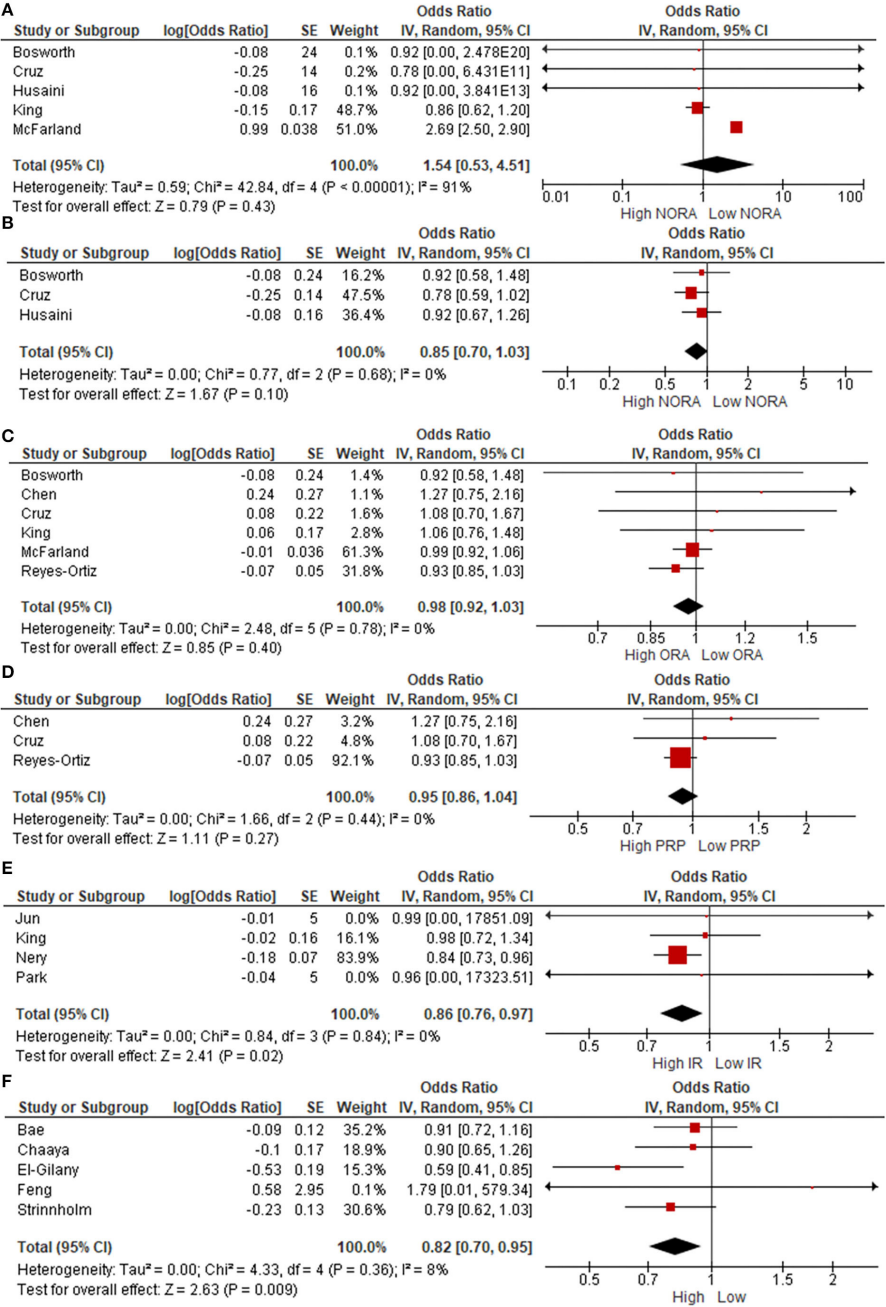
Odds Ratio (OR) for **(A)** NORA, **(B)** service attendance, **(C)** ORA, **(D)** private religious practices (PRP), **(E)** intrinsic religiosity, and **(F)** religious affiliation.

Figure 5 shows the cross-sectional association between RS and depressive symptoms according to binary data. No significant associations between NORA (OR = 1.54; 95 % CI = 0.53–4.51, *P* = 0.43; χ^2^ = 42.84, df = 4; I^2^ = 91%; *P* < 0.00001), service attendance (OR = 0.85; 95 % CI = 0.70–1.03, *P* = 0.10; χ^2^ = 0.77, df = 2; I^2^ = 0%; *P* = 0.68), ORA (OR = 0.98; 95 % CI = 0.92–1.03, *P* = 0.40; χ^2^ = 2.48, df = 5; I^2^ = 0%; *P* = 0.78), and private religious practice (OR = 0.95; 95 % CI = 0.86–1.04, *P* = 0.27; χ^2^ = 1.66, df = 2; I^2^ = 0%; *P* = 0.44) and depressive symptoms were observed. On the other hand, a lower prevalence of depressive symptoms was observed in older adults with high IR (OR = 0.86; 95 % CI = 0.76–0.97, *P* = 0.02; χ^2^ = 0.84, df = 3; I^2^ = 0%; *P* = 0.84) and those affiliated to any religion (OR = 0.82; 95 % CI = 0.70–0.95, *P* = 0.009; χ^2^ = 4.33, df = 4; I^2^ = 8%; *P* = 0.36).

#### Hopelessness

Three studies investigated the association between religious aspects and hopelessness. Menon et al. (127) observed a higher prevalence of hopelessness in male hospitalized older veterans with low religious levels. Cruz et al. (19) noted that prayer, but not church attendance, was significantly associated with a lower prevalence of hopelessness in older adults who attended geriatric inpatient units and outpatient clinics. More recently, Mitchell et al. (27) analyzed a cohort of 887 black men who participated in the Health and Retirement Study. The authors noted that participants with high religious attendance and religiosity had lower hopelessness levels.

#### Life Satisfaction

**Figure 6:**
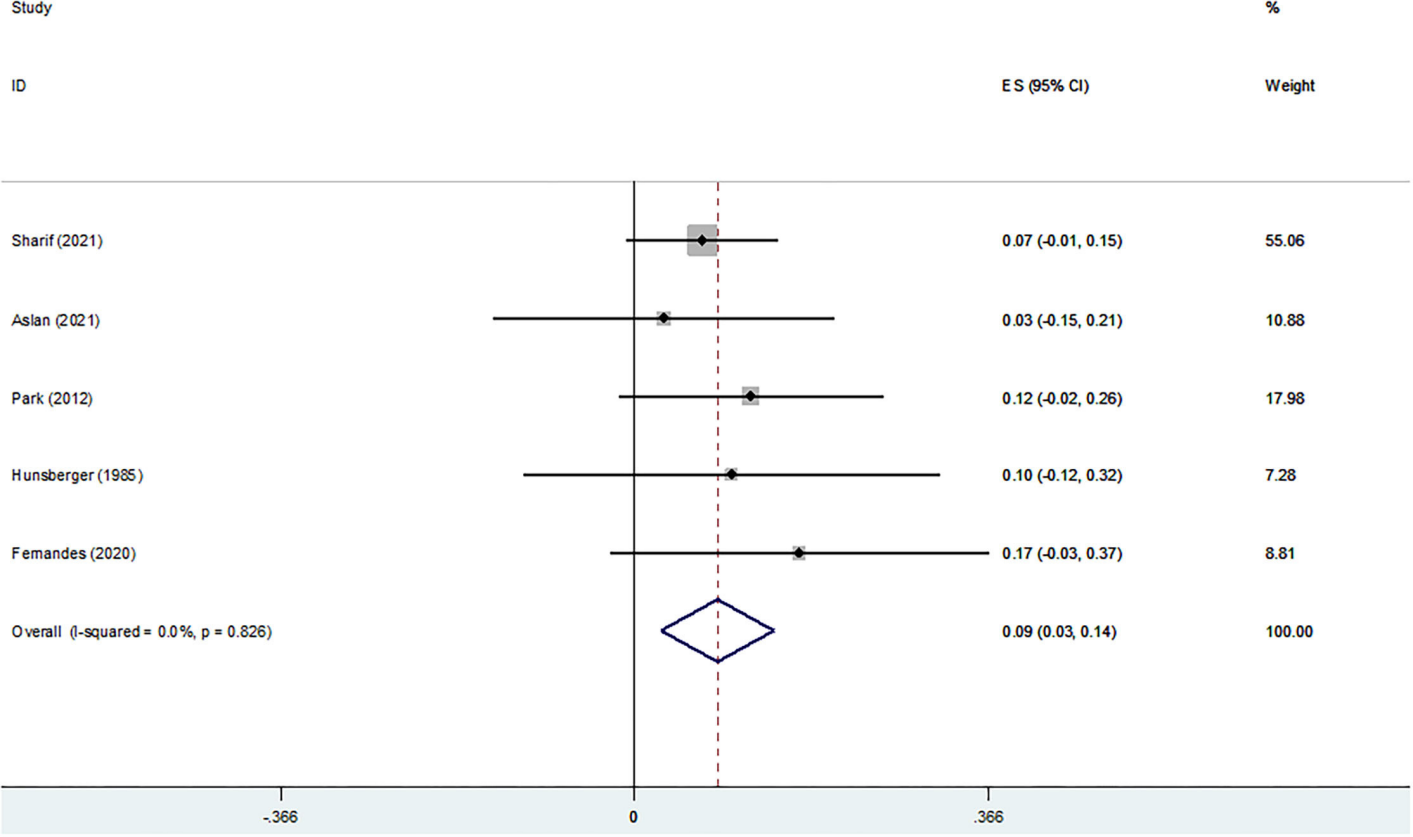
Z-scores for life satisfaction.

Figure 6 shows the cross-sectional association between life satisfaction and religiosity. The pooled analysis of five studies indicated a positive and significant association (Z-score = 0.086, 95 % CI = 0.027–0.144, *P* = 0.004).

#### Meaning in Life

**Figure 7:**
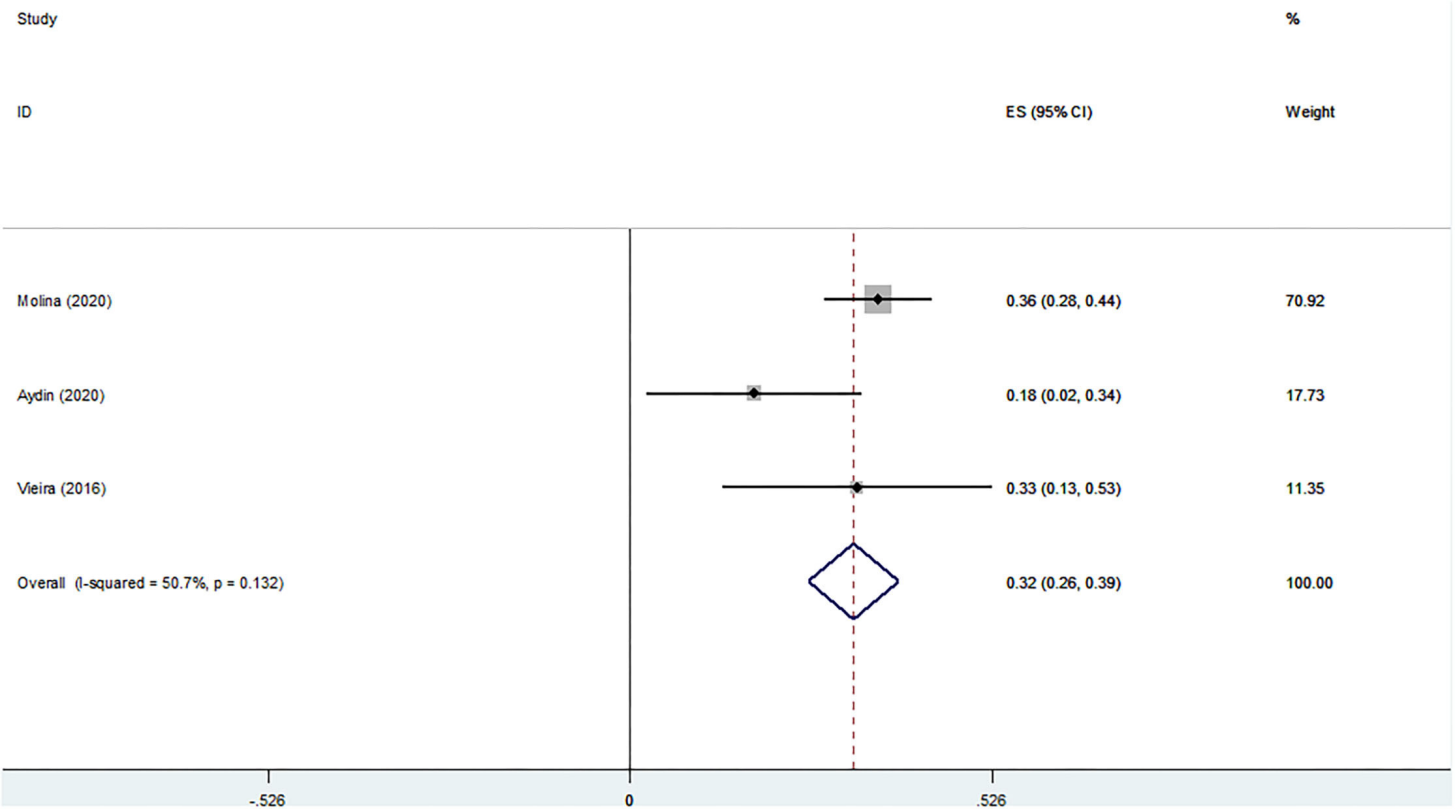
Z-scores for meaning in life.

Figure 7 shows the association between RS and meaning in life. The pooled analysis was conducted with three studies and the results indicated a significant association (Z-score = 0.325, 95 % CI = 0.259–0.391, *P* = 0.0001).

#### General Mental Health

Three studies investigated the association between RS and global mental health. Two studies assessed global mental health using the subscale of the SF-36 (160, 169), while one study used the subscale of the SF-12 (46). Meisenhelder and Chandler (160) reported a significant association between the frequency of prayer and the importance of faith with general mental in older native people. Parker et al. (46) noted a significant association between ORA, but not NORA and IR, and general mental health in community-dwelling older adults. Investigating older Korean immigrants, Lee et al. (169) reported that spiritual coping was negatively associated with general mental health. However, no significant relations were noted with private religious practice.

#### Psychological Well-Being

**Figure 8:**
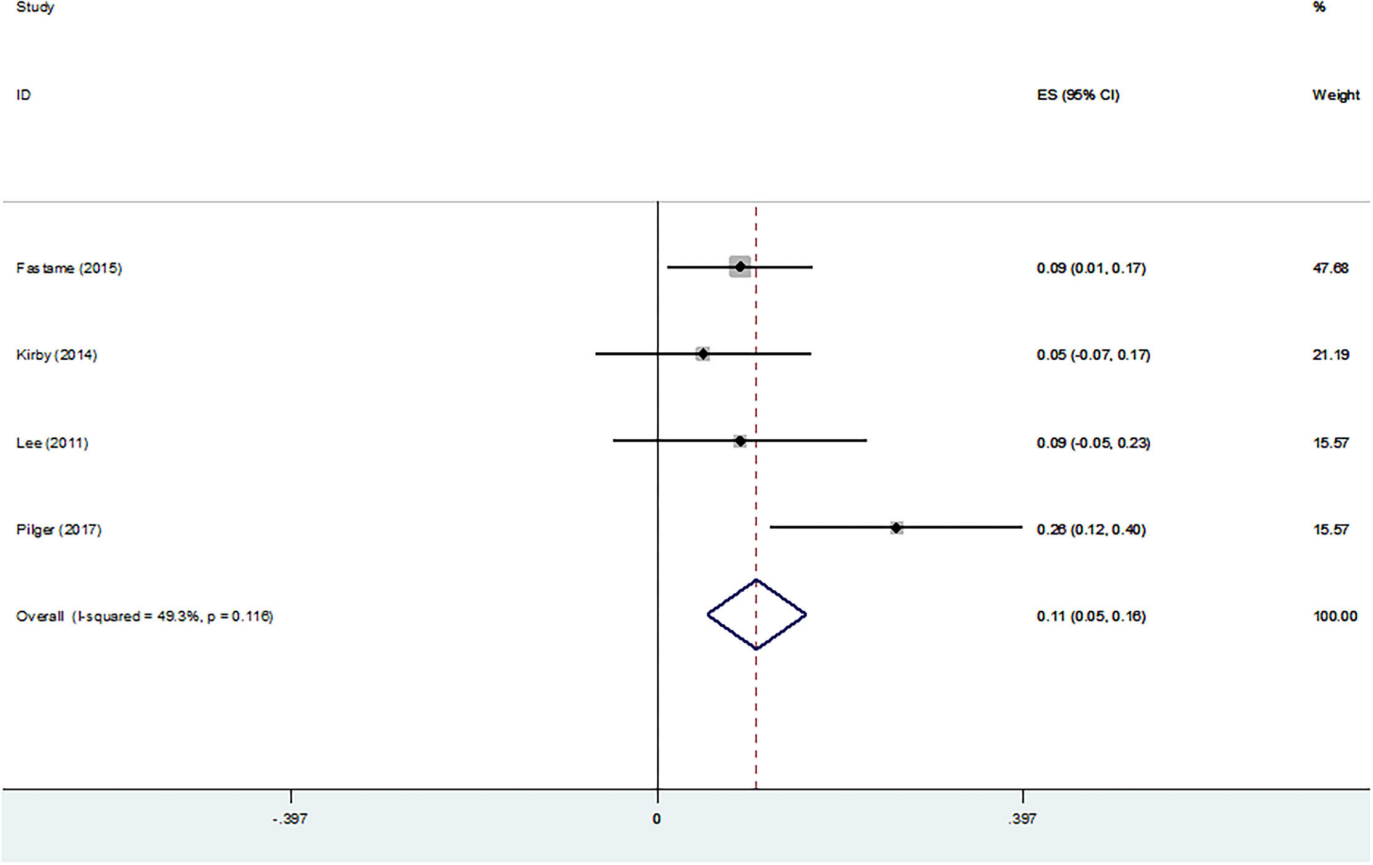
Z-scores for psychological well-being.

Figure 8 shows the association between psychological well-being and RS. A significant association was observed (Z-score = 0.108, 95 % CI = 0.054–0.162, *P* = 0.0001).

#### Self-Esteem

The association between self-esteem and RS was investigated in three studies. Guglani et al. (126) examined Asian Hindu grandmothers who lived in London and observed that religious participation was not significantly associated with self-esteem. Similar results were found by Krause et al. (133), who noted no effects of NORA and ORA on self-esteem. However, significant associations were observed with religious coping. Keyes et al. (113) supported these results by indicating that neither religious attendance nor religiosity, but religious identity, was associated with self-esteem.

#### Social Relations

**Figure 9:**
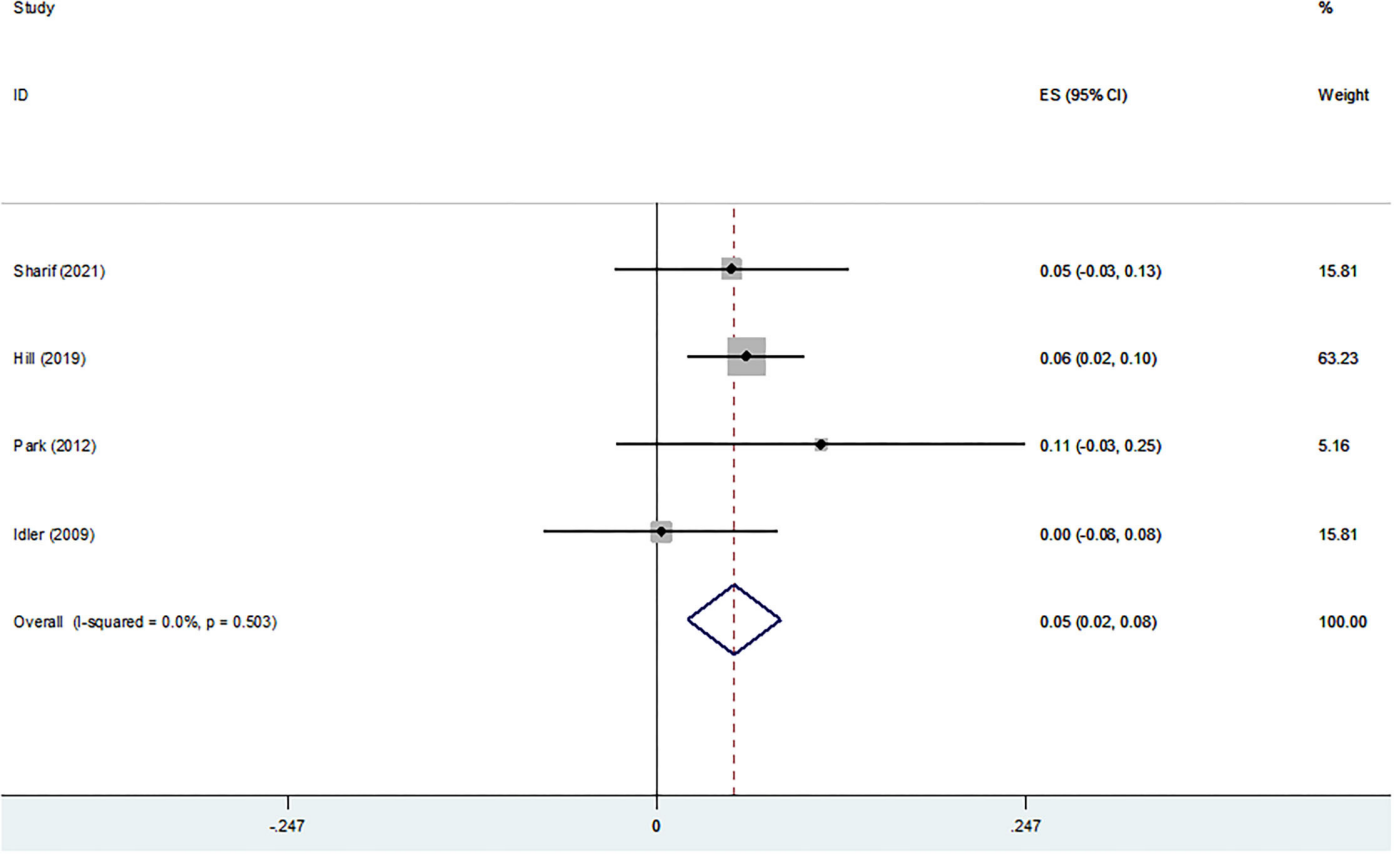
Z-scores for social relations.

Figure 9 shows the cross-sectional association between RS and social relations according to continuous data. The pooled analysis of four studies indicated a significant association (Z-score = 0.052, 95 % CI = 0.021–0.083, *P* = 0.001).

**Figure 10:**
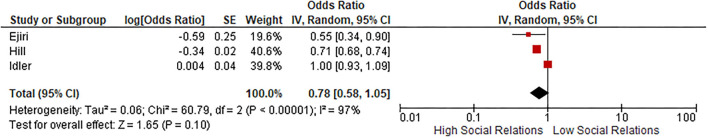
Odds Ratio (OR) for social relations.

Figure 10 shows the cross-sectional association between RS and social relations according to binary data. No significant associations between religious levels and social health were observed (OR = 0.78; 95 % CI = 0.58–1.05, *P* = 0.10; χ^2^ = 60.79, df = 2; I^2^ = 97%; *P* < 0.00001).

#### Suicidal Ideation

The association between suicidal ideation and RS was investigated in four studies. Rivera-Ledesma and Montero-Lòpez Lena (90) noted that vital spirituality and dissatisfaction were associated with suicidal ideation in Pentecostal Mexican older adults. Moreover, Bonnewyn et al. (82) identified that private religiousness was associated with the wish to die in patients admitted to a psychiatry department. In contrast, Jahn et al. (94) did not observe significant relations between DUREL scores and the Geriatric Suicide Ideation Scale (GSIS). These results were partially supported by Jung et al. (29), who observed no significant effects of NORA and ORA on suicidal ideation, although a negative association was observed with IR.

#### Tobacco Use

Three studies investigated the association between tobacco use and religiosity. Abdel-Hady and El-Gilany (59) reported that low religiosity increased the likelihood of tobacco smoking use in Egyptian older adults. Lee Roff et al. (117) conducted an analysis according to religious aspects and noted that both NORA and ORA were negatively associated with cigarette smoking. Similarly, Blay et al. (108) observed a lower prevalence of tobacco users in participants who had experienced religious changes, have religion as an orienting-motivating force, have any religious affiliation and high social religiosity.

#### Publication Bias

The funnel plot for depressive symptoms is shown in Supplementary Material 6. No publication bias was identified according to Egger's test (*P* = 0.836).

### Longitudinal

#### Death Anxiety

One study investigated the association between religiosity and death anxiety. Jung et al. (162) analyzed data from the Religion, Aging, and Health Survey and observed that secure attachment to God was associated with a lower risk of developing death anxiety 3 years later.

#### Depressive Symptoms

The longitudinal effects of religion on depressive symptoms were studied in six studies. Kivela et al. (168) investigated two waves (1984–1985 and 1989–1990) of Older Finns and noted that low religious participation increased the risk of depression in women, but not in men. These results were further expanded by Norton et al. (167), Law and Sbarra (166), Sun et al. (165), and Roh et al. (170) who observed that older adults with higher church attendance had a reduced risk of developing depression and depressive symptoms in a short-time-interval. Hui-Chuan (164) investigated the role of NORA on the severity of depressive symptoms over a 4-year follow-up period. Authors reported that prayer and reading the scriptures were associated with reduced depressive symptoms, whereas praying for calmness and praying for help were accompanied by a significant increase in depressive symptoms.

#### General Mental Health

One study investigated the association between religion and mental health. In this investigation, Ysseldyk et al. (92) conducted a 6-year follow-up and observed that religious identity was significantly associated with better mental health.

## Discussion

RS is a social phenomenon that might be expressed across a plethora of activities, depending on individuals' desires, beliefs, motivation, needs, accessibility, availability, and recognition, to quote a few variables. Participation and adherence to religious practices might influence numerous health-related parameters, including mental health. This scenario is important because older adults are commonly very religious and particularly susceptible to mental health problems. However, until now, no prior investigations have provided a clearly evidence-based scenario on the association between RS and mental health in older adults.

Our results indicate that RS is significantly associated with many mental health parameters, including anxiety, depressive symptoms, life satisfaction, meaning in life, social relations, and psychological well-being. In addition, investigations provided encouraging longitudinal results regarding the effects of RS on death anxiety, depressive symptoms, and general mental health.

### Cross-Sectional and Longitudinal Associations Between RS and Mental Health Aspects

Results of the current study indicate that RS was negatively associated with anxiety. Numerous RS-related parameters might contribute to the attenuation of anxiety symptoms. Generalized and social anxiety, for example, are associated with loneliness in community-dwelling older adults (171, 172). In contrast, ORA is expected to provide a favorable environment for expanding the social network and creating intimate relationships. Experts in the field have suggested that religious communities might serve as an extension of the biological family (173, 174). Increased self-care is another possible candidate to explain the current results, given that anxiety is significantly associated with the prevalence of chronic diseases (175), whereas RS doctrine and philosophy are frequently concentrated on healthy lifestyle habits, avoiding the excessive consumption of tobacco, alcohol, and food (47). Notably, anxiety and depressive symptoms are usually observed simultaneously in older patients since they share many clinical symptoms and psychosocial and neurobiological mechanisms (176, 177). Hence, the possibility that changes in anxiety are secondary to improvements in depressive symptoms might not be ruled out. However, specific religious parameters were not investigated in the included studies hindering a better understanding of this scenario.

We observed that RS parameters were negatively associated with depressive symptoms. Particularly, older adults with high spirituality had fewer depressive symptoms in comparison to their counterparts. Furthermore, IR and religious affiliation were negatively associated with the prevalence of depressive symptoms. These results were supported by longitudinal studies, which reported that more religious and spiritual people had a lower risk of developing depression and worsening depressive symptoms. Taken together, these data indicate that approaching life according to the belief in the presence of the Divine (e.g., nature, energy, God) might be negatively associated with depressive symptoms in older adults.

The possibility that RS activities might contribute to coping with adverse life events has long been discussed in the literature, and many possible mediators have been suggested to explain this association. Giving meaning to suffering, for instance, can provide a cognitive framework to understand and manage negative life experiences (37, 178). Numerous religions explain suffering as an opportunity for purification, learning, and personal growth, in an attempt to escape from punishment and obtain awards in the current and/or in the afterlife (37, 178). This perspective was exhaustively discussed by Weber (14), who pointed out that this mechanism is similarly observed in the major religious groups worldwide.

RS might also reduce depressive symptoms by providing guidance and support in moments of frustration and despair. This scenario is based on the perception that the Divine is taking care of peoples' lives and that everything occurs according to a better purpose, even if sometimes humans cannot understand God's plan (37, 178). This scenario was well-illustrated in the discourse of a 61-year-old Peruvian Woman examined by Flores-Flores et al. (179), who explained that *God* was her *psychologist* and that she *prayed to him every night to take* her *negative feelings away from her*, and, according to her, *little by little, they went away* (179). In other cases, believing in the occurrence of spiritual experiences seemed to provide comfort and conciliation. Alaska native older adults who have experienced grieving mentioned that they constantly feel the presence of dead relatives by seeing lights or hearing voices and that these events contribute to coping with the losses (180).

Religious groups might also provide guidance and support by providing instructions of how people can go through difficult times and how religious communities can give support in these moments (37). The Jewish tradition is a good illustration of this scenario by describing structured, graduated, and linear periods by which people are likely to pass through in the course of mourning (181). Such perspective takes into consideration the numerous feelings that might be experienced during this period, and indicate the tasks that might be conducted by familiars to cope with the stages of grief (181). The Jewish community, in its turn, is advised about the best moments in which they can provide comfort and companionship (181).

Giving meaning to suffering is another potential mechanism by which RS can reduce depressive symptomatology (49, 178). Finding meaning in life is recognized as an important element in the maintenance of mental health by offering coherence in peoples' lives. This concept has been incorporated as a key component of the theoretical bases of many psychological schools. In logotherapy, the third Viennese school of psychotherapy, Frankl (182) proposed that finding meaning in life is the primary motor force of human beings. This perspective describes that people must take control and be responsible for all decisions concerning their lives, instead of acting passively, regardless of the circumstances. In contrast, the inability to identify meaning in life might trigger the development of numerous mental health problems, including depression. A complementary view is provided by Erikson (183), on his theory of psychosocial development. According to him, older adults that are unable to give meaning to their accomplishments in life tend to become desperate, hopeless, and depressive (183).

These premises also provide light to justify the significant association between death anxiety and religiosity (162), given that failure to understand life's meaning may produce a psychologically stressful environment that promotes an increase in death anxiety (184, 185). This point of view was explored by Becker (186) in the death deny theory. It describes that finding meaning in life has a vital role as a symbolic defense against the notion of mortality and consequently death anxiety (184, 185).

Finally, religious and spiritualist people might live their lives according to a transcendent approach, reducing the excessive focus on the self and changing the attention to other people with worse suffering (178). Focus on other people's well-being can increase the feeling of connection with high power and serve as a life purpose (178). Such attitude might impact depressive symptoms, given the close association between personality traits with a high self-conscious component and depression (187).

It is important to note that numerous other parameters might have impacted the negative association between IR and depression, such as NORA and ORA. However, the cross-sectional design of the included studies prevents us from making strong assumptions and points out the need for longitudinal studies.

Another major finding of the present study is that RS was significantly associated with numerous positive feelings, including life satisfaction, meaning in life, social relations, and psychological well-being. As no specific RS elements (e.g., NORA, ORA, IR) were investigated it is hard to suggest the main mechanisms underlying these associations. Nevertheless, social relations are predominately influenced by the feeling of belonging, worship and mutual spiritual support produced by ORA (188). In contrast, life satisfaction and meaning in life are affected by many variables, such as social, affective and psychological aspects (189).

Psychological well-being, in its turn, is a large multidimensional construct that dependents on the combination of emotional status, personality traits, and life experiences (188). Prior studies have suggested that RS might influence psychological well-being by modulating perceived control (190), positive emotions (191), social support (96), and coping mechanisms (192, 193). Furthermore, the impact of numerous indirect mechanisms associated with self-care might not be ruled out (41, 47).

### Clinical Implications and Directions for Future Research

Older patients' management is commonly based on pharmacological therapy and gerontologists have claimed that understanding the role of RS beliefs on peoples' health might contribute to better and more qualified care (194). Such criticism is based on the fact that older adults have mentioned that they would feel better if health professionals responsible for their care tried to include their personal beliefs in the treatment (48). However, most health professionals do not have basic knowledge in this field (48), although it might be essential in some contexts. Hospitalized patients, for example, often ask if their disease is a divine punishment and whether God will take care of them (195).

Our findings encourage health professionals to understand patients' beliefs and search for the best method to include RS elements in the therapeutic approach. Notably, NORA and ORA had a small direct impact on mental health parameters, whereas IR substantially influenced depressive symptomatology. These results might indicate that RS-based approaches to managing depressive symptoms in older adults should preferably allow patients to internalize, reflect, discuss, and solidify their personal beliefs than stimulate the adherence to religious events and the practice of private religious activities. In other cases, where was not possible to identify which RS elements were associated with better mental health status, such as anxiety, health professionals should try to distinguish which activities would make older adults feel more confident and comfortable.

The present study also detected important aspects that must be considered for future research. A fundamental observation is that most investigations have assessed RS using general scores and have not taken into consideration the importance of individual dimensions. This scenario limits the creation of more specific recommendations for health professionals responsible for the care of older adults. On the other hand, numerous dimensions have been tested against depressive symptoms, allowing us to indicate the most associated parameters. Hence, our results must be tested in large randomized clinical trials comparing RS-based treatments organized according to different dimensions (e.g., IR vs. NORA/ORA).

Another interesting point is that data from the systematic review suggested that most cross-sectional investigations showed that RS might be negatively associated with alcohol intake, tobacco use, and hopelessness. These findings are encouraging and suggest that RS might be a larger role in mental health status than those reported in the current study. However, the small number of evidence impeded us to conduct pooled and stratification analyses followed by heterogeneity and publication bias verification. In addition, not all investigations supported significant associations among the variables, indicating that conclusions must be taken carefully and that more studies are still necessary.

### Limitations

Numerous limitations restrict our findings. First, the majority of studies have a cross-sectional design and causality cannot be inferred. Second, results were predominately based on multidimensional scores and did not take into consideration specific RS elements. Third, the limited number of investigations impeded us to conduct pooled analysis for longitudinal studies and investigate publication bias and the sources of heterogeneity in other variables than depressive symptoms. Fourth, Z-scores were obtained by converting Pearson's correlation scores, which were not corrected according to possible covariables. Fifth, OR and Z-scores were not converted into a unique ES. Although we could have conducted this analysis using statistical methods, experts in the field have suggested that it is associated with a high risk of bias and might provide misleading results, mainly when studies provide low power and similar rates of cases and controls (51). Sixth, some ES were estimated by conversions. Seventh, many studies did not perform statistical regressions according to important covariables, including disease severity, drug treatment, physical activity levels, and dietary habits. Eight, the possible differences in mental health parameters among specific religious affiliations were not investigated. This aspect is important because a high prevalence of mental health problems has been observed in some religious contexts (196) due to the presence of rigid thinking, overdependence on laws and rules, and an emphasis on guilt and sin (37). Ninth, RS and mental health parameters were operationalized using different methods and they may have capture different dimensions. Tenth, we combined studies that have investigated alcohol and tobacco use and abuse. Eleventh, most studies involved community-dwelling older adults and our findings should be carefully extrapolated to hospitalized, institutionalized, and native people (119). Seventh, the present study investigated mental health parameters, and future studies are necessary to explore the association between RS with brain disorders and diseases. Finally, spirituality was poorly investigated.

## Conclusions

Our findings suggest that RS activities are significantly associated with mental health in older adults. Specifically, people with high RS levels had lower anxiety and depressive symptoms, as well as higher life satisfaction, meaning in life, social relations, and psychological well-being. Data provided by an increasing number of longitudinal studies have supported most of these findings.

## Data Availability Statement

The original contributions presented in the study are included in the article/Supplementary Material, further inquiries can be directed to the corresponding author.

## Author Contributions

HC-J, RC, FP, RA, AP, EM, and VA: conceived of the present idea and results interpretation. HC-J and RC: articles' search, qualitative analysis, and quantitative analysis. EM and VA: supervision. All authors discussed the results and commented on the manuscript.

## Funding

This work was partially funded by an Intramural Research Grant from the Università Cattolica del Sacro Cuore (EM), the Fundação de Apoio a Pesquisa do Distrito Federal (FAPDF), and the nonprofit research foundation Centro Studi Achille e Linda Lorenzon (to AP, EM, HC-J, and RC).

## Conflict of Interest

The authors declare that the research was conducted in the absence of any commercial or financial relationships that could be construed as a potential conflict of interest.

## Publisher's Note

All claims expressed in this article are solely those of the authors and do not necessarily represent those of their affiliated organizations, or those of the publisher, the editors and the reviewers. Any product that may be evaluated in this article, or claim that may be made by its manufacturer, is not guaranteed or endorsed by the publisher.
