# Supplementary material for: Religiosity/Spirituality and Mental Health in Older Adults: A Systematic Review and Meta-Analysis of Observational Studies
Source: Front Med (Lausanne). 2022 May 12;9:877213. doi: 10.3389/fmed.2022.877213 (PMC9133607; doi:10.3389/fmed.2022.877213)
Supplement: Supplementary file 1 [file Data_Sheet_1.docx]

**SM1 — Consulted Systematic and Non-Systematic Reviews and Meta-Analyses**

1. Braam, A. W., & Koenig, H. G. (2019). Religion, spirituality and depression in prospective studies: A systematic review. Journal of Affective Disorders, 257, 428-438.
2. Dein, S. (2006). Religion, spirituality and depression: Implications for research and treatment. Primary Care & Community Psychiatry.
3. Glas, G., & Poort, Z. (2007). Anxiety, anxiety disorders, religion and spirituality. Southern medical journal, 100(6), 621-625.
4. Koenig, H. G. (1990). Research on religion and mental health in later life: a review and commentary. Journal of Geriatric Psychiatry.
5. Koenig, H. G. (2009). Research on religion, spirituality, and mental health: A review. The Canadian Journal of Psychiatry, 54(5), 283-291.
6. Koenig, H. G. (2014). Depression in chronic illness: does religion help?. Journal of Christian Nursing, 31(1), 40-46.
7. Levin, J. (2010). Religion and mental health: Theory and research. International Journal of Applied Psychoanalytic Studies, 7(2), 102-115.
8. McCullough, M. E., & Larson, D. B. (1999). Religion and depression: A review of the literature. Twin Research and Human Genetics, 2(2), 126-136.
9. Rosmarin, D. H., & Leidl, B. (2020). Spirituality, religion, and anxiety disorders. Handbook of spirituality, religion, and mental health, 41-60.
10. Schieman, S., Bierman, A., & Ellison, C. G. (2013). Religion and mental health. In Handbook of the sociology of mental health (pp. 457-478). Springer, Dordrecht.
11. Shreve-Neiger, A. K., & Edelstein, B. A. (2004). Religion and anxiety: A critical review of the literature. Clinical psychology review, 24(4), 379-397.
