# Supplementary material for: Religiosity/Spirituality and Mental Health in Older Adults: A Systematic Review and Meta-Analysis of Observational Studies
Source: Front Med (Lausanne). 2022 May 12;9:877213. doi: 10.3389/fmed.2022.877213 (PMC9133607; doi:10.3389/fmed.2022.877213)
Supplement: Supplementary file 2 [file Data_Sheet_2.docx]

**PUBMED**

**Participant**

Older adults

Elderly

**Intervention**

African Religions

Anglicanism

Assyrians

Buddhism

Candomble

Catholicism

Christianity

Confucianism

Eastern Orthodoxy

Hinduism

Islam

Jainism

Judaism

Oriental Orthodoxy

Protestantism

Religion [MESH]

Religiosity

Religious Beliefs

Sikhism

Spiritism

Spirituality

Taoism

Umbanda

**Outcomes**

Alcohol Drinking [MESH]

Anxiety [MESH]

Anxiety Disorders [MESH]

Chemical Dependence

Depression [MESH]

Depressive Disorder [MESH]

Depressive Symptoms

Distress

Drug Abuse

Drug Addiction

Drug Dependence

Dyssomnias [MESH]

Emotional Depression

Emotional exhaustion

Fear [MESH]

Insomnia

Mental Disorders [MESH]

Mental Fatigue [MESH]

Mental Health [MESH]

Mental Hygiene

Mental Illness

Obsession-Compulsion

Panic Attack

Panic Disorder [TIAB]

Phobia

Phobic Disorders [MESH]

Psychological Burnout [MESH]

Psychological Distress [MESH]

Psychological stress

Self-harm

Self-Injurious Behavior [MESH]

Sleep disorder

Sleep Initiation and Maintenance Disorders [MESH]

Stress-Related Disorders

Substance Abuses

Substance Use Disorders

Substance-Related Disorders [MESH]

Suicide [MESH]
