# Supplementary material for: Religiosity/Spirituality and Mental Health in Older Adults: A Systematic Review and Meta-Analysis of Observational Studies
Source: Front Med (Lausanne). 2022 May 12;9:877213. doi: 10.3389/fmed.2022.877213 (PMC9133607; doi:10.3389/fmed.2022.877213)
Supplement: Supplementary file 3 [file Data_Sheet_3.docx]

**EXCLUDED**

**Age**

1. A. W., Beekman, A. T., Deeg, D. J., Smit, J. H., & van Tilburg, W. (1997). Religiosity as a protective or prognostic factor of depression in later life; results from a community survey in The Netherlands. Acta Psychiatrica Scandinavica, 96(3), 199-205.
2. Aday, R. H., Krabill, J. J., & Deaton-Owens, D. (2014). Religion in the lives of older women serving life in prison. Journal of Women & Aging, 26(3), 238-256.
3. Ahrenfeldt, L. J., Möller, S., Andersen-Ranberg, K., Vitved, A. R., Lindahl-Jacobsen, R., & Hvidt, N. C. (2017). Religiousness and health in Europe. European journal of epidemiology, 32(10), 921-929.
4. Ai, A. L., Peterson, C., Bolling, S. F., & Rodgers, W. (2006). Depression, faith-based coping, and short-term postoperative global functioning in adult and older patients undergoing cardiac surgery. Journal of Psychosomatic Research, 60(1), 21-28.
5. Allen, R. S., Harris, G. M., Crowther, M. R., Oliver, J. S., Cavanaugh, R., & Phillips, L. L. (2013). Does religiousness and spirituality moderate the relations between physical and mental health among aging prisoners?. International journal of geriatric psychiatry, 28(7), 710-717.
6. Baeke, G., Wils, J. P., & Broeckaert, B. (2012). “Be Patient and Grateful”-Elderly Muslim Women's Responses to Illness and Suffering. Journal of Pastoral Care & Counseling, 66(3), 1-9.
7. Balbuena, L., Baetz, M., & Bowen, R. (2013). Religious attendance, spirituality, and major depression in Canada: A 14-year follow-up study. The Canadian Journal of Psychiatry, 58(4), 225-232.
8. Bishop, A. J., Randall, G. K., & Merten, M. J. (2014). Consideration of forgiveness to enhance the health status of older male prisoners confronting spiritual, social, or emotional vulnerability. Journal of Applied Gerontology, 33(8), 998-1017.
9. Boswell, G. H., Kahana, E., & Dilworth-Anderson, P. (2006). Spirituality and healthy lifestyle behaviors: Stress counter-balancing effects on the well-being of older adults. Journal of Religion and Health, 45(4), 587-602.
10. Braam, A. W., Beekman, A. T. F., Van Tilburg, T. G., Deeg, D. J. H., & Van Tilburg, W. (1997). Religious involvement and depression in older Dutch citizens. Social Psychiatry and Psychiatric Epidemiology, 32(5), 284-291.
11. Braam, A. W., Hein, E., Deeg, D. J., Twisk, J. W., Beekman, A. T., & Van Tilburg, W. (2004). Religious involvement and 6-year course of depressive symptoms in older Dutch citizens: results from the Longitudinal Aging Study Amsterdam. Journal of Aging and Health, 16(4), 467-489.
12. Brandão, B. M. G. D. M., Angelim, R. C. D. M., Marques, S. C., Oliveira, R. C. D., & Abrão, F. M. D. S. (2020). Living with HIV: coping strategies of seropositive older adults. Revista da Escola de Enfermagem da USP, 54.
13. Caliari, J. D. S., Reinato, L. A. F., Pio, D. P. M., Lopes, L. P., Reis, R. K., & Gir, E. (2018). Quality of life of elderly people living with HIV/AIDS in outpatient follow-up. Revista brasileira de enfermagem, 71, 513-522.
14. Chatters, L. M., Bullard, K. M., Taylor, R. J., Woodward, A. T., Neighbors, H. W., & Jackson, J. S. (2008). Religious participation and DSM-IV disorders among older African Americans: findings from the National Survey of American Life. The American Journal of Geriatric Psychiatry, 16(12), 957-965.
15. Chen, Y. Y., & Koenig, H. G. (2006). Do people turn to religion in times of stress?: An examination of change in religiousness among elderly, medically ill patients. The Journal of nervous and mental disease, 194(2), 114-120.
16. Cohen, C. I., Jimenez, C., & Mittal, S. (2010). The role of religion in the well-being of older adults with schizophrenia. Psychiatric Services, 61(9), 917-922.
17. Commerford, M. C., & Reznikoff, M. (1996). Relationship of religion and perceived social support to self-esteem and depression in nursing home residents. The Journal of psychology, 130(1), 35-50.
18. Cook, J. M., Pearson, J. L., Thompson, R., Black, B. S., & Rabins, P. V. (2002). Suicidality in older African Americans: findings from the EPOCH study. The American Journal of Geriatric Psychiatry, 10(4), 437-446.
19. Despoina, M., Marianna, T., Moses, G., & Magda, T. (2018). Differences in religiosity among cognitively intact, mildly cognitively impaired, and mildly demented elderly, and its possible relationship with depressive mood. Applied Nursing Research, 43, 69-74.
20. Duarte, F. M., & Wanderley, K. D. S. (2011). Religião e espiritualidade de idosos internados em uma enfermaria geriátrica. Psicologia: teoria e pesquisa, 27, 49-53.
21. Emlet, C. A., Harris, L., Pierpaoli, C. M., & Furlotte, C. (2018). “The journey i have been through”: The role of religion and spirituality in aging well among HIV-positive older adults. Research on aging, 40(3), 257-280.
22. Grodensky, C. A., Golin, C. E., Jones, C., Mamo, M., Dennis, A. C., Abernethy, M. G., & Patterson, K. B. (2015). “I should know better”: The roles of relationships, spirituality, disclosure, stigma, and shame for older women living with HIV seeking support in the South. Journal of the Association of Nurses in AIDS Care, 26(1), 12-23.
23. Hamilton, J. B., Sandelowski, M., Moore, A. D., Agarwal, M., & Koenig, H. G. (2013). “You need a song to bring you through”: The use of religious songs to manage stressful life events. The Gerontologist, 53(1), 26-38.
24. Huang, C. Y., Hsu, M. C., & Chen, T. J. (2012). An exploratory study of religious involvement as a moderator between anxiety, depressive symptoms and quality of life outcomes of older adults. Journal of clinical nursing, 21(5‐6), 609-619.
25. Hunter, B. D., & Merrill, R. M. (2013). Religious orientation and health among active older adults in the United States. Journal of religion and health, 52(3), 851-863.
26. Ironson, G., Lucette, A., Hylton, E., Pargament, K. I., & Krause, N. (2018). The relationship between religious and psychospiritual measures and an inflammation marker (CRP) in older adults experiencing life event stress. Journal of religion and health, 57(4), 1554-1566.
27. Joarder, T., Cooper, A., & Zaman, S. (2014). Meaning of death: an exploration of perception of elderly in a Bangladeshi village. Journal of cross-cultural gerontology, 29(3), 299-314.
28. Kim, H. H. S., & Jung, J. H. (2021). Ageism, religiosity, and wellbeing among older adults: evidence from the European Social Survey (ESS4). Research on Aging, 43(5-6), 214-226.
29. Kodzi, I. A., Gyimah, S. O., Emina, J., & Ezeh, A. C. (2011). Religious involvement, social engagement, and subjective health status of older residents of informal neighborhoods of Nairobi. Journal of Urban Health, 88(2), 370-380.
30. Koenig, H. G. (2007). Religion and depression in older medical inpatients. The American journal of geriatric psychiatry, 15(4), 282-291.
31. Koenig, H. G., George, L. K., & Titus, P. (2004). Religion, spirituality, and health in medically ill hospitalized older patients. Journal of the American Geriatrics society, 52(4), 554-562.
32. Krause, N., & Van Tran, T. (1989). Stress and religious involvement among older blacks. Journal of Gerontology, 44(1), S4-S13.
33. Krause, N., Ingersoll-Dayton, B., Ellison, C. G., & Wulff, K. M. (1999). Aging, religious doubt, and psychological well-being. The Gerontologist, 39(5), 525-533.
34. Lamba, N., Bhatia, A., Shrivastava, A., & Raghavan, A. (2021). Religious factors affecting death anxiety in older adults practicing Hinduism. Death Studies, 1-9.
35. Leavey, G., Loewenthal, K., & King, M. (2007). Challenges to sanctuary: The clergy as a resource for mental health care in the community. Social science & medicine, 65(3), 548-559.
36. Leelavanichkul, S., Chamratrithirong, A., Jampaklay, A., & Gray, R. (2018). Religiosity, the practices of religions, and the perception of older people among Muslims and Buddhists in Thailand. The International Journal of Aging and Human Development, 86(2), 131-151.
37. Leurent, B., Nazareth, I., Bellón-Saameño, J., Geerlings, M. I., Maaroos, H., Saldivia, S., ... & King, M. (2013). Spiritual and religious beliefs as risk factors for the onset of major depression: an international cohort study. Psychological medicine, 43(10), 2109-2120.
38. Levin, J. S., & Chatters, L. M. (1998). Religion, health, and psychological well-being in older adults: Findings from three national surveys. Journal of aging and health, 10(4), 504-531.
39. Lewis, J. P., & Allen, J. (2017). Alaska Native elders in recovery: Linkages between indigenous cultural generativity and sobriety to promote successful aging. Journal of Cross-Cultural Gerontology, 32(2), 209-222.
40. Marques, F. D., Sousa, L. M., Vizzotto, M. M., & Bonfim, T. E. (2015). A vivência dos mais velhos em uma comunidade indígena Guarani Mbyá. Psicologia & Sociedade, 27, 415-427.
41. McCauley, J., Tarpley, M. J., Haaz, S., & Bartlett, S. J. (2008). Daily spiritual experiences of older adults with and without arthritis and the relationship to health outcomes. Arthritis Care & Research: Official Journal of the American College of Rheumatology, 59(1), 122-128.
42. Mitchell, B. D., Lee, W. J., Tolea, M. I., Shields, K., Ashktorab, Z., Magder, L. S., ... & Schäffer, A. A. (2012). Living the good life? Mortality and hospital utilization patterns in the Old Order Amish. PloS one, 7(12), e51560.
43. Mölsä, M., Kuittinen, S., Tiilikainen, M., Honkasalo, M. L., & Punamäki, R. L. (2017). Mental health among older refugees: the role of trauma, discrimination, and religiousness. Aging & Mental Health, 21(8), 829-837.
44. Morton, K. R., Lee, J. W., Haviland, M. G., & Fraser, G. E. (2012). Religious engagement in a risky family model predicting health in older Black and White Seventh-Day Adventists. Psychology of religion and spirituality, 4(4), 298.
45. Nelson-Becker, H. (2005). Religion and coping in older adults: A social work perspective. Journal of Gerontological Social Work, 45(1-2), 51-67.
46. O'Brien, B., Shrestha, S., Stanley, M. A., Pargament, K. I., Cummings, J., Kunik, M. E., ... & Amspoker, A. B. (2019). Positive and negative religious coping as predictors of distress among minority older adults. International journal of geriatric psychiatry, 34(1), 54-59.
47. Ofstedal, M. B., Chiu, C. T., Jagger, C., Saito, Y., & Zimmer, Z. (2019). Religion, life expectancy, and disability-free life expectancy among older women and men in the United States. The Journals of Gerontology: Series B, 74(8), e107-e118.
48. Park, C. L., Malone, M. R., Suresh, D. P., Bliss, D., & Rosen, R. I. (2008). Coping, meaning in life, and quality of life in congestive heart failure patients. Quality of Life Research, 17(1), 21-26.
49. Pokorski, M., & Warzecha, A. (2011). Depression and religiosity in older age. European journal of medical research, 16(9), 401-406.
50. Rivera-Ledesma, A., & Montero-López Lena, M. (2007). Medidas de afrontamiento religioso y espiritualidad en adultos mayores mexicanos. Salud mental, 30(1), 39-47.
51. Roh, S., Brown-Rice, K. A., Lee, K. H., Lee, Y. S., Lawler, M. J., & Martin, J. I. (2015). Stressors, coping resources, and depressive symptoms among rural American Indian older adults. Social Work in Public Health, 30(4), 345-359.
52. Ronneberg, C. R., Miller, E. A., Dugan, E., & Porell, F. (2016). The protective effects of religiosity on depression: A 2-year prospective study. The Gerontologist, 56(3), 421-431.
53. Rosik, C. H. (1989). The impact of religious orientation in conjugal bereavement among older adults. The International Journal of Aging and Human Development, 28(4), 251-260.
54. Rosmarin, D. H., Malloy, M. C., & Forester, B. P. (2014). Spiritual struggle and affective symptoms among geriatric mood disordered patients. International journal of geriatric psychiatry, 29(6), 653-660.
55. Rusa, S. G., Peripato, G. I., Pavarini, S. C. I., Inouye, K., Zazzetta, M. S., & Orlandi, F. D. S. (2015). Quality of life/spirituality, religion and personal beliefs of adult and elderly chronic kidney patients under hemodialysis1. Revista latino-americana de enfermagem, 22, 911-917.
56. Rushing, N. C., Corsentino, E., Hames, J. L., Sachs-Ericsson, N., & Steffens, D. C. (2013). The relationship of religious involvement indicators and social support to current and past suicidality among depressed older adults. Aging & Mental Health, 17(3), 366-374.
57. Sasson, S. (2001). Religiosity as a factor affecting adjustment of minority elderly to a nursing home. Social Thought, 20(3-4), 77-96.
58. Schuurmans-Stekhoven, J. B. (2019). Auspicious or suspicious—Does religiosity really promote elder well-being? Examining the belief-as-benefit effect among older Japanese. Archives of gerontology and geriatrics, 81, 129-135.
59. Stanley, M. A., Bush, A. L., Camp, M. E., Jameson, J. P., Phillips, L. L., Barber, C. R., ... & Cully, J. A. (2011). Older adults’ preferences for religion/spirituality in treatment for anxiety and depression. Aging & mental health, 15(3), 334-343.
60. Stearns, M., Nadorff, D. K., Lantz, E. D., & McKay, I. T. (2018). Religiosity and depressive symptoms in older adults compared to younger adults: Moderation by age. Journal of affective disorders, 238, 522-525.
61. Stolz, E., Fux, B., Mayerl, H., Rásky, É., & Freidl, W. (2016). Passive suicide ideation among older adults in Europe: a multilevel regression analysis of individual and societal determinants in 12 countries (SHARE). Journals of Gerontology Series B: Psychological Sciences and Social Sciences, 71(5), 947-958.
62. Van Herreweghe, L., & Van Lancker, W. (2019). Is religiousness really helpful to reduce depressive symptoms at old age? A longitudinal study. Plos one, 14(7), e0218557.
63. Vance, D. E., Struzick, T. C., & Raper, J. L. (2008). Biopsychosocial benefits of spirituality in adults aging with HIV: Implications for nursing practice and research. Journal of Holistic Nursing, 26(2), 119-125.
64. Williams, B. J. (2008). An exploratory study of older adults' perspectives of spirituality. Occupational Therapy in Health Care, 22(1), 3-19.
65. Wink, P., & Scott, J. (2005). Does religiousness buffer against the fear of death and dying in late adulthood? Findings from a longitudinal study. The Journals of Gerontology Series B: Psychological Sciences and Social Sciences, 60(4), P207-P214.
66. Yeager, D. M., Glei, D. A., Au, M., Lin, H. S., Sloan, R. P., & Weinstein, M. (2006). Religious involvement and health outcomes among older persons in Taiwan. Social Science & Medicine, 63(8), 2228-2241.

**Did Not Assess/Study Mental Health**

1. Alencar, D. L. D., Marques, A. P. D. O., Leal, M. C. C., & Vieira, J. D. C. M. (2016). The exercise of sexuality among the elderly and associated factors. Revista Brasileira de Geriatria e Gerontologia, 19, 861-869.
2. Bailly, N., Martinent, G., Ferrand, C., Agli, O., Giraudeau, C., Gana, K., & Roussiau, N. (2018). Spirituality, social support, and flexibility among older adults: a five-year longitudinal study. International psychogeriatrics, 30(12), 1745-1752.
3. Betancur, A. M. H. (2013). Bienestar espiritual de adultos mayores con enfermedades crónicas de la ciudad de Santa Marta, Colombia. Cultura del cuidado, 10(1), 27-37.
4. Bowen, C. E., & Luy, M. (2018). Community social characteristics and health at older ages: evidence from 156 religious communities. The Journals of Gerontology: Series B, 73(8), 1429-1438.
5. Bowles, J., Brooks, T., Hayes-Reams, P., Butts, T., Myers, H., Allen, W., & Kington, R. S. (2000). Frailty, family, and church support among urban African American elderly. Journal of Health Care for the Poor and Underserved, 11(1), 87-99.
6. Bulsing, R. S., & Jung, S. I. (2016). Envelhecimento e morte: percepção de idosas de um grupo de convivência. Psicologia em Estudo, 21(1), 89-100.
7. Cagle, J. G., LaMantia, M. A., Williams, S. W., Pek, J., & Edwards, L. J. (2016). Predictors of preference for hospice care among diverse older adults. American Journal of Hospice and Palliative Medicine®, 33(6), 574-584.
8. Coats, H., Crist, J. D., Berger, A., Sternberg, E., & Rosenfeld, A. G. (2017). African American elders’ serious illness experiences: narratives of “God did,”“God will,” and “life is better”. Qualitative health research, 27(5), 634-648.
9. Costa, M. S., Moreira, M. A. S. P., Silva, A. O., Leite, E. D. S., Silva, L. M., & Sampaio, J. B. (2018). Knowledge, beliefs, and attitudes of older women in HIV/AIDS prevention. Revista brasileira de enfermagem, 71, 40-46.
10. Costaa, R., Carreraa, M., & Marquesa, A. P. FATORES QUE INFLUENCIAM A QUALIDADE DE VIDA GLOBAL DE IDOSOS LONGEVOS. Geriatr Gerontol Aging. 2021;15:e0210002
11. Croezen, S., Avendano, M., Burdorf, A., & Van Lenthe, F. J. (2015). Social participation and depression in old age: a fixed-effects analysis in 10 European countries. American journal of epidemiology, 182(2), 168-176.
12. Daaleman, T. P., Perera, S., & Studenski, S. A. (2004). Religion, spirituality, and health status in geriatric outpatients. The Annals of Family Medicine, 2(1), 49-53.
13. de Mello, J. G., Gresele, A. D., Maria, C. M., & Fedosse, E. (2013). Subjetividade e institucionalização no discurso de idosas. Distúrbios da Comunicação, 25(1).
14. Gonçalves, J. D. S., Silva, L. L. D., Abdala, G. A., Meira, M. D. D., Santos, A. C. M., & Silva, M. D. F. F. D. (2017). Religiosidade e os transtornos mentais comuns em adultos. Rev. enferm. UFPE on line, 1708-1715.
15. Hamren, K., Chungkham, H. S., & Hyde, M. (2015). Religion, spirituality, social support and quality of life: measurement and predictors CASP-12 (v2) amongst older Ethiopians living in Addis Ababa. Aging & mental health, 19(7), 610-621.
16. Heeren, O., Menon, A. S., Raskin, A., & Ruskin, P. (2001). Religion and end of life treatment preferences among geriatric patients. International journal of geriatric psychiatry, 16(2), 203-208.
17. Herlina, A. (2019). Spirituality and health status among elderly people in nursing home in Riau, Indonesia. Enfermeria Clinica, 29, 5-13.
18. Ingersoll-Dayton, B., Torges, C., & Krause, N. (2010). Unforgiveness, rumination, and depressive symptoms among older adults. Aging & mental health, 14(4), 439-449.
19. Iveniuk, J., O’Muircheartaigh, C., & Cagney, K. A. (2016). Religious influence on older Americans’ sexual lives: A nationally-representative profile. Archives of sexual behavior, 45(1), 121-131.
20. Koenig, H. G. (1998). Religious attitudes and practices of hospitalized medically ill older adults. International journal of geriatric psychiatry, 13(4), 213-224.
21. Koenig, H. G., Moberg, D. O., & Kvale, J. N. (1988). Religious activities and attitudes of older adults in a geriatric assessment clinic. Journal of the American Geriatrics Society, 36(4), 362-374.
22. Krause, N., Ingersoll-Dayton, B., Liang, J., & Sugisawa, H. (1999). Religion, social support, and health among the Japanese elderly. Journal of health and social behavior, 405-421.
23. Markides, K. S. (1983). Aging, religiosity, and adjustment: A longitudinal analysis. Journal of Gerontology, 38(5), 621-625.
24. Moura, W. C. S. D. (2015). Consciência da finitude e valores humanos: um estudo com idosos em instituições de longa permanência. Estud. interdiscipl. envelhec., v. 23, n. 3, p. 9-25.
25. Musick, M. A. (1996). Religion and subjective health among black and white elders. Journal of Health and Social behavior, 221-237.
26. Ocampo, J. M., Romero, N., Saa, H. A., Herrera, J. A., & Reyes-Ortiz, C. A. (2006). Prevalencia de las prácticas religiosas, disfunción familiar, soporte social y síntomas depresivos en adultos mayores. Cali, Colombia 2001. Colombia Médica, 37(2), 26-30.
27. Ohr, S., Jeong, S., & Saul, P. (2017). Cultural and religious beliefs and values, and their impact on preferences for end‐of‐life care among four ethnic groups of community‐dwelling older persons. Journal of Clinical Nursing, 26(11-12), 1681-1689.
28. Okun, M. A., O’Rourke, H. P., Keller, B., Johnson, K. A., & Enders, C. (2015). Value-expressive volunteer motivation and volunteering by older adults: Relationships with religiosity and spirituality. Journals of Gerontology Series B: Psychological Sciences and Social Sciences, 70(6), 860-870.
29. Opsahl, T., Kørup, A. K., Andersen-Ranberg, K., Christensen, K., & Hvidt, N. C. (2021). Characteristics of Danish Centenarians’ Religious Beliefs: A Nationwide Population-Based Study. Journal of religion and health, 60(3), 2007-2023.
30. Pickard, J. G. (2006). The relationship of religiosity to older adults’ mental health service use. Aging and Mental Health, 10(3), 290-297.
31. Reiling, D. M. (2002). Boundary maintenance as a barrier to mental health help‐seeking for depression among the Old Order Amish. The Journal of Rural Health, 18(3), 428-436.
32. Reyes-Ortiz, C. A., Payan, C., Altamar, G., Gomez, F., & Koenig, H. G. (2019). Religiosity and self-rated health among older adults in Colombia. Colombia Médica, 50(2), 67-76.
33. Reyes-Ortiz, C. A., Pelaez, M., Koenig, H. G., & Mulligan, T. (2007). Religiosity and self-rated health among Latin American and Caribbean elders. The International Journal of Psychiatry in Medicine, 37(4), 425-443.
34. Richardson, T. M., Friedman, B., Podgorski, C., Knox, K., Fisher, S., He, H., & Conwell, Y. (2012). Depression and its correlates among older adults accessing aging services. The American journal of geriatric psychiatry, 20(4), 346-354.
35. Salamene, L. C., Martins, E. L. M., Lucchetti, G., & Lucchetti, A. L. G. (2021). Factors associated with successful aging in Brazilian community-dwelling older adults: When physical health is not enough. Geriatric Nursing, 42(2), 372-378.
36. Silva, A. T. D. M., Tavares, D. M. D. S., Molina, N. P. F. M., Assunção, L. M. D., & Rodrigues, L. R. (2019). Religiosidade e espiritualidade relacionadas às variáveis sociodemográficas, econômicas e de saúde entre idosos. Revista Mineira de Enfermagem, 23, 1-7.
37. Smith-Cavros, E., Avotri-Wuaku, J., Wuaku, A., & Bhullar, A. (2017). “All I need is help to do well”: herbs, medicines, faith, and syncretism in the negotiation of elder health treatment in rural Ghana. Journal of religion and health, 56(6), 2129-2143.
38. Taylor, R. J., & Chatters, L. M. (1991). Nonorganizational religious participation among elderly black adults. Journal of Gerontology, 46(2), S103-S111.
39. Taylor, R. J., Chatters, L. M., & Joe, S. (2011). Non-organizational religious participation, subjective religiosity, and spirituality among older African Americans and Black Caribbeans. Journal of religion and health, 50(3), 623-645.
40. Taylor, R. J., Chatters, L. M., McKeever Bullard, K., Wallace Jr, J. M., & Jackson, J. S. (2009). Organizational religious behavior among older African Americans: findings from the national survey of American life. Research on aging, 31(4), 440-462.
41. Vicente, A. R. T., Castro-Costa, É., Firmo, J. D. O. A., Lima-Costa, M. F., & Loyola, A. I. D. (2018). Religiousness, social support and the use of antidepressants among the elderly: a population-based study. Ciencia & saude coletiva, 23, 963-971.
42. Waddell, E. L., & Jacobs-Lawson, J. M. (2010). Predicting positive well-being in older men and women. The International Journal of Aging and Human Development, 70(3), 181-197.
43. Yang, C., Ford, M. E., Tilley, B. C., & Greene, R. L. (2016). Religiosity in black and white older Americans: Measure adaptation, psychometric validation, and racial difference. Medicine, 95(37).
44. Zenevicz, L., Moriguchi, Y., & Madureira, V. S. F. (2013). The religiosity in the process of living getting old. Revista da Escola de Enfermagem da USP, 47, 433-439.
45. Zorn, C. R., & Johnson, M. T. (1997). Religious well‐being in noninstitutionalized elderly women. Health Care for Women International, 18(3), 209-219.

**Did Not Assess/Study Religiosity and/or Spirituality**

1. Baptista, A. S. D., Neves, S. T. V. D., & Baptista, M. N. (2008). Correlação entre suporte familiar, saúde mental e crenças irracionais em idosos religiosos. Psic: revista da Vetor Editora, 9(2), 155-164.
2. McKechnie, J., & Hill, E. M. (2009). Alcoholism in older women religious. Substance Abuse, 30(2), 107-117.
3. Salma, J., & Salami, B. (2020). “Growing Old is not for the Weak of Heart”: Social isolation and loneliness in Muslim immigrant older adults in Canada. Health & social care in the community, 28(2), 615-623.
4. Shemesh, A. A., Kohn, R., Blumstein, T., Geraisy, N., Novikov, I., & Levav, I. (2006). A community study on emotional distress among Arab and Jewish Israelis over the age of 60. International Journal of Geriatric Psychiatry: A journal of the psychiatry of late life and allied sciences, 21(1), 64-76.
5. Soonthornchaiya, R., & Dancy, B. L. (2006). Perceptions of depression among elderly Thai immigrants. Issues in Mental Health Nursing, 27(6), 681-698.
6. Van Nguyen, T., Van Nguyen, H., Nguyen, T. D., Van Nguyen, T., & Nguyen, T. T. (2017). Difference in quality of life and associated factors among the elderly in rural Vietnam. Journal of Preventive Medicine and Hygiene, 58(1), E63.

**Did Not Assess the Association Between Mental Health and Religiosity/Spirituality**

1. Adib-Hajbaghery, M., & Faraji, M. (2015). Comparison of happiness and spiritual well-being among the community dwelling elderly and those who lived in sanitariums. International journal of community based nursing and midwifery, 3(3), 216.
2. Ahn, S., Phillips, K. L., Smith, M. L., & Ory, M. G. (2011). Correlates of volunteering among aging Texans: The roles of health indicators, spirituality, and social engagement. Maturitas, 69(3), 257-262.
3. Alano, G. J., Pekmezaris, R., Tai, J. Y., Hussain, M. J., Jeune, J., Louis, B., ... & Wolf-Klein, G. P. (2010). Factors influencing older adults to complete advance directives. Palliative & Supportive Care, 8(3), 267-275.
4. Alonso Palacio, L. M., Ríos, A. L., Caro de Payares, S., Maldonado, A., Campo, L., Quiñonez, D., & Zapata, Y. (2010). Percepción del envejecimiento y bienestar que tienen los adultos mayores del Hogar Geriátrico San Camilo de la ciudad de Barranquilla (Colombia). Revista Salud Uninorte, 26(2), 250-259.
5. Ayele, H., Mulligan, T., Gheorghiu, S., & Reyes‐Ortiz, C. (1999). Religious activity improves life satisfaction for some physicians and older patients. Journal of the American Geriatrics Society, 47(4), 453-455.
6. Barlev, M., Mermelstein, S., Cohen, A. S., & German, T. C. (2019). The embodied God: Core intuitions about person physicality coexist and interfere with acquired Christian beliefs about God, the Holy Spirit, and Jesus. Cognitive science, 43(9), e12784.
7. Bilotta, C., Bowling, A., Casè, A., Nicolini, P., Mauri, S., Castelli, M., & Vergani, C. (2010). Dimensions and correlates of quality of life according to frailty status: a cross-sectional study on community-dwelling older adults referred to an outpatient geriatric service in Italy. Health and quality of life outcomes, 8(1), 1-10.
8. Bradshaw, M., Ellison, C. G., Fang, Q., & Mueller, C. (2015). Listening to religious music and mental health in later life. The Gerontologist, 55(6), 961-971.
9. Brandão, V. C., & Zatt, G. B. (2015). Percepção de idosos, moradores de uma instituição de longa permanência de um município do interior do Rio Grande do Sul, sobre qualidade de vida. Aletheia, (46).
10. Buono, M. D., Urciuoli, O., & LEO, D. D. (1998). Quality of life and longevity: a study of centenarians. Age and ageing, 27(2), 207-216.
11. Carmel, S., & Mutran, E. (1997). Preferences for different life-sustaining treatments among elderly persons in Israel. The Journals of Gerontology Series B: Psychological Sciences and Social Sciences, 52(2), S97-S102.
12. Castellanos Soriano, F., & López Díaz, A. L. (2010). Mirando pasar la vida desde la ventana: significados de la vejez y la discapacidad de un grupo de ancianos en un contexto de Pobreza. Investig. enferm, 37-53.
13. Chen, H. C., Chan, S. W. C., Yeh, T. P., Huang, Y. H., Chien, I. C., & Ma, W. F. (2019). The Spiritual Needs of Community‐Dwelling Older People Living With Early‐Stage Dementia—A Qualitative Study. Journal of Nursing Scholarship, 51(2), 157-167.
14. Cheng, S. T., Chen, P. P., Mok, M. H., Chow, Y. F., Chung, J. W., Law, A. C., ... & Tam, C. W. (2020). Typology of pain coping and associations with physical health, mental health, and pain profiles in Hong Kong Chinese older adults. Aging & Mental Health, 1-9.
15. Choi, N. G., An, S., & DiNitto, D. M. (2021). Felt age among racial/ethnic minority older adults attending a senior center. Journal of Applied Gerontology, 40(4), 395-403.
16. Cicirelli, V. G. (1997). Relationship of psychosocial and background variables to older adults' end-of-life decisions. Psychology and Aging, 12(1), 72.
17. Die, A. H., & Seelbach, W. C. (1988). Problems, sources of assistance, and knowledge of services among elderly Vietnamese immigrants. The Gerontologist, 28(4), 448-452.
18. Dunn, K. S., & Horgas, A. L. (2000). The prevalence of prayer as a spiritual self-care modality in elders. Journal of Holistic Nursing, 18(4), 337-351.
19. Elo, S., Saarnio, R., & Isola, A. (2011). The physical, social and symbolic environment supporting the well-being of home-dwelling elderly people. International Journal of Circumpolar Health, 70(1), 90-100.
20. Fairrow, A. M., McCallum, T. J., & Messinger-Rapport, B. J. (2004). Preferences of older African-Americans for long-term tube feeding at the end of life. Aging & mental health, 8(6), 530-534.
21. Foong, H. F., Hamid, T. A., Ibrahim, R., & Haron, S. A. (2018). Moderating effect of intrinsic religiosity on the relationship between depression and cognitive function among community-dwelling older adults. Aging & mental health, 22(4), 483-488.
22. Gallardo-Peralta, L. P., & Sánchez-Moreno, E. (2019). Successful Aging and Personal Well-Being Among the Chilean Indigenous and Non-Indigenous Elderly. Aquichan, 19(3).
23. Gerwood, J. B., LeBlanc, M., & Piazza, N. (1998). The purpose‐in‐life test and religious denomination: Protestant and Catholic scores in an elderly population. Journal of Clinical Psychology, 54(1), 49-53.
24. Griffin, M. T. Q., Lee, Y. H., Salman, A., Seo, Y., Marin, P. A., Starling, R. C., & Fitzpatrick, J. J. (2007). Spirituality and well being among elders: differences between elders with heart failure and those without heart failure. Clinical interventions in aging, 2(4), 669.
25. Hodge, D. R., & Wolosin, R. (2014). Spiritual needs and satisfaction with service provision: Mediating pathways among a national sample of hospital inpatients. Social work research, 38(3), 135-143.
26. Hodge, D. R., & Wolosin, R. J. (2015). Failure to address African Americans’ spiritual needs during hospitalization: Identifying predictors of dissatisfaction across the arc of service provision. Journal of gerontological social work, 58(2), 190-205.
27. Huijer, H. A. S., Bejjani, R., & Fares, S. (2019). Quality of care, spirituality, relationships and finances in older adult palliative care patients in Lebanon. Annals of palliative medicine, 8(5), 551-558.
28. Kaufmann, C. N., Montross-Thomas, L. P., & Griser, S. (2018). Increased engagement with life: Differences in the cognitive, physical, social, and spiritual activities of older adult music listeners. The Gerontologist, 58(2), 270-277.
29. Kim, S. S., & Kim-Godwin, Y. S. (2019). Cultural Context of Family Religiosity/Spirituality among Korean-American Elderly Families. Journal of cross-cultural gerontology, 34(1), 51-65.
30. Kim, S. S., Hayward, R. D., & Kang, Y. (2013). Psychological, physical, social, and spiritual well-being similarities between Korean older adults and family caregivers. Geriatric Nursing, 34(1), 35-40.
31. Kim, S. S., Kim-Godwin, Y. S., & Koenig, H. G. (2016). Family spirituality and family health among Korean-American elderly couples. Journal of religion and health, 55(2), 729-746.
32. Koenig, H. G., George, L. K., & Peterson, B. L. (1998). Religiosity and remission of depression in medically ill older patients. American Journal of Psychiatry, 155(4), 536-542.
33. Laird, K. T., Lavretsky, H., Paholpak, P., Vlasova, R. M., Roman, M., Cyr, N. S., & Siddarth, P. (2019). Clinical correlates of resilience factors in geriatric depression. International psychogeriatrics, 31(2), 193-202.
34. Levin, J. S., Markides, K. S., & Ray, L. A. (1996). Religious attendance and psychological well-being in Mexican Americans: A panel analysis of three-generations data. The Gerontologist, 36(4), 454-463.
35. Litwin, H. (1998). Social network type and health status in a national sample of elderly Israelis. Social science & medicine, 46(4-5), 599-609.
36. LOWENSTEIN, S. A. S. A. (1997). Suicide among the elderly in Israel. Death studies, 21(4), 361-375.
37. Manasatchakun, P., Chotiga, P., Roxberg, Å., & Asp, M. (2016). Healthy ageing in Isan-Thai culture—a phenomenographic study based on older persons’ lived experiences. International Journal of Qualitative studies on Health and Well-being, 11(1), 29463.
38. Maragh-Bass, A. C., Sloan, D. H., Alghanim, F., & Knowlton, A. R. (2021). A mixed-methods exploration of faith, spirituality, and health program interest among older African Americans with HIV. Quality of Life Research, 30(2), 507-519.
39. McFarland, M. J., Uecker, J. E., & Regnerus, M. D. (2011). The role of religion in shaping sexual frequency and satisfaction: Evidence from married and unmarried older adults. Journal of Sex Research, 48(2-3), 297-308.
40. Nahapetyan, L., Orpinas, P., Glass, A., & Song, X. (2019). Planning ahead: Using the theory of planned behavior to predict older adults’ intentions to use hospice if faced with terminal illness. Journal of Applied Gerontology, 38(4), 572-591.
41. O'Connor, B. P., & Vallerand, R. J. (1990). Religious motivation in the elderly: A French-Canadian replication and an extension. The Journal of Social Psychology, 130(1), 53-59.
42. Pickard, J. G., & Guo, B. (2008). Clergy as mental health service providers to older adults. Aging and Mental Health, 12(5), 615-624.
43. Putney, J. M., Leafmeeker, R. R., & Hebert, N. (2016). “The Wisdom of Age”: Perspectives on aging and growth among lesbian older adults. Journal of gerontological social work, 59(3), 234-251.
44. Rahemi, Z. (2019). Planning ahead for end-of-life healthcare among Iranian-American older adults: attitudes and communication of healthcare wishes. Journal of cross-cultural gerontology, 34(2), 187-199.
45. Richmond, R. L., Law, J., & Kay‐Lambkin, F. (2011). Physical, mental, and cognitive function in a convenience sample of centenarians in Australia. Journal of the American Geriatrics Society, 59(6), 1080-1086.
46. Rosati, F., Pistella, J., Nappa, M. R., & Baiocco, R. (2020). The coming-out process in family, social, and religious contexts among young, middle, and older Italian LGBQ+ adults. Frontiers in Psychology, 11, 3481.
47. Sawchuk, D. (2015). Aging and older adults in three Roman Catholic magazines: Successful aging and the Third and Fourth Ages reframed. Journal of aging studies, 35, 221-228.
48. Scelzo, A., Di Somma, S., Antonini, P., Montross, L. P., Schork, N., Brenner, D., & Jeste, D. V. (2018). Mixed-methods quantitative–qualitative study of 29 nonagenarians and centenarians in rural Southern Italy: focus on positive psychological traits. International psychogeriatrics, 30(1), 31-38.
49. Shellman, J. (2004). “Nobody ever asked me before”: understanding life experiences of African American elders. Journal of Transcultural Nursing, 15(4), 308-316.
50. Silva, G. O., Peixoto, L. C. P., Souza, D. A. D., Santos, A. L. D. S., & Aguiar, A. C. D. S. A. (2018). Repercussões do adoecimento crônico na saúde mental de pessoas idosas. Rev. enferm. UFPE on line, 2923-2932.
51. Teixeira, J. J. V., & Lefèvre, F. (2008). Significado da intervenção médica e da fé religiosa para o paciente idoso com câncer. Ciência & Saúde Coletiva, 13, 1247-1256.
52. Vitorino, L. M., Low, G., & Lucchetti, G. (2019). Is the physical environment associated with spiritual and religious coping in older age? Evidence from Brazil. Journal of religion and health, 58(5), 1648-1660.
53. Walls, C. T., & Zarit, S. H. (1991). Informal support from black churches and the well-being of elderly blacks. The Gerontologist, 31(4), 490-495.
54. Wang, W. T., He, B., Wang, Y. H., Wang, M. Y., Chen, X. F., Wu, F. C., & Yang, X. (2017). The relationships among Muslim Uyghur and Kazakh disabled elders' life satisfaction, activity of daily living, and informal family caregiver's burden, depression, and life satisfaction in far western China: A structural equation model. International journal of nursing practice, 23(2), e12521.
55. Washington, O. G., Moxley, D. P., Garriott, L., & Weinberger, J. P. (2009). Five dimensions of faith and spiritually of older African American women transitioning out of homelessness. Journal of Religion and Health, 48(4), 431-444.
56. Williams, G. L., Keigher, S., & Williams, A. V. (2012). Spiritual well-being among older African Americans in a midwestern city. Journal of religion and health, 51(2), 355-370.
57. Wortman, E. S., & Lewis, J. P. (2021). Gerotranscendence and Alaska Native Successful Aging in the Aleutian Pribilof Islands, Alaska. Journal of Cross-Cultural Gerontology, 36(1), 43-67.
58. Zamorano, A., Muñoz, M., Ausín, B., & Pérez, E. (2019). Relación entre la salud mental y el nivel de funcionamiento de las personas mayores de 65 años de la comunidad de madrid. Clínica y Salud, 30(2), 63-71.

**Review**

1. Gallagher, D. E., Thompson, L. W., & Peterson, J. A. (1982). Psychosocial factors affecting adaptation to bereavement in the elderly. The International Journal of Aging and Human Development, 14(2), 79-95.
2. Vance, D. E., Brennan, M., Enah, C., Smith, G. L., & Kaur, J. (2011). Religion, spirituality, and older adults with HIV: Critical personal and social resources for an aging epidemic. Clinical Interventions in Aging, 6, 101.

**Psychometric Evaluation, Validity or Translation**

1. Bush, A. L., Jameson, J. P., Barrera, T., Phillips, L. L., Lachner, N., Evans, G., ... & Stanley, M. A. (2012). An evaluation of the brief multidimensional measure of religiousness/spirituality in older patients with prior depression or anxiety. Mental Health, Religion & Culture, 15(2), 191-203.

**Lack of information**

1. Blinderman, C. D., Homel, P., Billings, J. A., Portenoy, R. K., & Tennstedt, S. L. (2008). Symptom distress and quality of life in patients with advanced congestive heart failure. Journal of pain and symptom management, 35(6), 594-603.
2. Ellison, C. G., & Flannelly, K. J. (2009). Religious involvement and risk of major depression in a prospective nationwide study of African American adults. The Journal of Nervous and Mental Disease, 197(8), 568-573.
3. Gitlin, L. N., Hauck, W. W., Dennis, M. P., & Schulz, R. (2007). Depressive symptoms in older African‐American and white adults with functional difficulties: the role of control strategies. Journal of the American Geriatrics Society, 55(7), 1023-1030.
4. Park, C. L., Lim, H., Newlon, M., Suresh, D. P., & Bliss, D. E. (2014). Dimensions of religiousness and spirituality as predictors of well-being in advanced chronic heart failure patients. Journal of Religion and Health, 53(2), 579-590.
5. Park, C. L., Wortmann, J. H., & Edmondson, D. (2011). Religious struggle as a predictor of subsequent mental and physical well-being in advanced heart failure patients. Journal of Behavioral Medicine, 34(6), 426-436.
