# Supplementary material for: Religiosity/Spirituality and Mental Health in Older Adults: A Systematic Review and Meta-Analysis of Observational Studies
Source: Front Med (Lausanne). 2022 May 12;9:877213. doi: 10.3389/fmed.2022.877213 (PMC9133607; doi:10.3389/fmed.2022.877213)
Supplement: Supplementary file 4 [file Data_Sheet_4.docx]

| **Supplementary Material 4**. Quality analysis | | | | | | | | | | | | | | | | |
| --- | --- | --- | --- | --- | --- | --- | --- | --- | --- | --- | --- | --- | --- | --- | --- | --- |
|  | **Religious** |  |  |  |  |  |  |  |  |  |  |  |  |  |  |  |
| *Cross-sectional* | | | | | | | | | | | | | | | | |
| **Year** | **Authors** | **1** | **2** | **3** | **4** | **5** | **6** | **7** | **8** | **9** | **10** | **11** | **12** | **13** | **14** | **Overall score (0/10)** |
| *Religiosity* | | | | | | | | | | | | | | | | |
| 2020 | Aslan et al. | Y | Y | Y | Y | N | NA | NA | Y | Y | N | Y | NR | NA | N | 7 |
| 2020 | Foong et al. | Y | Y | Y | Y | N | NA | NA | Y | Y | N | Y | NR | NA | Y | 8 |
| 2020 | Bae | Y | Y | Y | Y | N | NA | NA | N | Y | N | Y | NR | NA | N | 6 |
| 2020 | Abdel-Hady & El-Gilany | Y | Y | Y | Y | Y | NA | NA | N | Y | N | Y | NR | NA | N | 7 |
| 2020 | Mitchell et al. | Y | Y | Y | Y | N | NA | NA | N | Y | N | Y | NR | NA | Y | 7 |
| 2020 | Molina et al. | Y | Y | NR | Y | Y | NA | NA | N | Y | N | Y | NR | NA | N | 6 |
| 2020 | Sharif et al. | Y | Y | N | Y | Y | NA | NA | N | Y | N | Y | NR | NA | Y | 7 |
| 2020 | Solaimanizadeh et al. | Y | Y | NR | N | N | NA | NA | Y | Y | N | Y | NR | NA | N | 5 |
| 2020 | Gallardo-Peralta and Sánchez-Moreno | Y | Y | NR | N | N | NA | NA | N | Y | N | Y | NR | NA | Y | 5 |
| 2019 | Silva et al. | Y | Y | NR | Y | N | NA | NA | N | Y | N | Y | NR | NA | N | 5 |
| 2019 | Bakhtiari et al. | Y | Y | NR | Y | N | NA | NA | Y | Y | N | Y | NR | NA | N | 6 |
| 2019 | Bakan et al. | Y | Y | N | N | N | NA | NA | N | Y | N | Y | NR | NA | Y | 5 |
| 2019 | Fernández-Niño et al. | Y | Y | Y | Y | N | NA | NA | N | Y | N | Y | NR | NA | Y | 7 |
| 2019 | Hamid et al. | Y | Y | Y | Y | N | NA | NA | Y | Y | N | Y | NR | NA | Y | 8 |
| 2019 | Hill et al. | Y | Y | Y | Y | N | NA | NA | N | Y | N | Y | NR | NA | Y | 7 |
| 2019 | Reyes-Ortiz et al. | Y | Y | Y | Y | Y | NA | NA | Y | Y | N | Y | NR | NA | N | 8 |
| 2019 | Strinnholm et al. | Y | Y | Y | Y | N | NA | NA | Y | Y | N | Y | NR | NA | N | 7 |
| 2019 | Willis et al. | Y | Y | NR | Y | N | NA | NA | Y | Y | N | Y | NR | NA | N | 6 |
| 2019 | Moreno et al. | Y | Y | NR | N | N | NA | NA | N | Y | N | Y | NR | NA | N | 4 |
| 2019 | Ejiri et al. | Y | Y | Y | Y | N | NA | NA | N | Y | N | Y | NR | NA | Y | 7 |
| 2018 | El-Gilany et al. | Y | Y | Y | Y | Y | NA | NA | N | Y | N | Y | NR | NA | N | 7 |
| 2018 | Kotian et al. | Y | Y | N | N | N | NA | NA | N | Y | N | Y | NR | NA | N | 4 |
| 2018 | Manning and Miles | Y | Y | NR | Y | Y | NA | NA | N | Y | N | Y | NR | NA | N | 6 |
| 2018 | Nery et al. | Y | Y | N | Y | N | NA | NA | Y | Y | N | Y | NR | NA | N | 6 |
| 2018 | Munawar and Tariq | Y | Y | NR | N | N | NA | NA | N | Y | N | Y | NR | NA | N | 4 |
| 2017 | Jung et al. | Y | Y | N | Y | N | NA | NA | Y | Y | N | Y | NR | NA | N | 6 |
| 2017 | Lac et al. | Y | Y | NR | Y | N | NA | NA | Y | Y | N | Y | NR | NA | N | 6 |
| 2017 | Nunes et al. | Y | Y | N | Y | N | NA | NA | N | Y | N | Y | NR | NA | N | 5 |
| 2016 | Bonnewyn et al. | Y | Y | Y | Y | N | NA | NA | Y | Y | N | Y | NR | NA | Y | 8 |
| 2016 | McGowan et al. | Y | Y | NR | N | N | NA | NA | Y | Y | N | Y | NR | NA | Y | 6 |
| 2016 | Krok | Y | Y | NR | N | N | NA | NA | Y | Y | N | Y | NR | NA | N | 5 |
| 2016 | Vieira and Aquino | Y | Y | NR | N | N | NA | NA | Y | Y | N | Y | NR | NA | N | 5 |
| 2015 | Abdala et al. | Y | Y | NR | N | N | NA | NA | Y | Y | N | Y | NR | NA | Y | 6 |
| 2015 | Fastame et al. | Y | Y | Y | Y | N | NA | NA | N | Y | N | Y | NR | NA | N | 6 |
| 2015 | Stecz and Kocur | Y | Y | Y | N | N | NA | NA | Y | Y | N | Y | NR | NA | N | 6 |
| 2014 | Andrade | Y | Y | NR | Y | Y | NA | NA | N | Y | N | Y | NR | NA | N | 6 |
| 2014 | Feng et al. | Y | Y | Y | Y | N | NA | NA | N | Y | N | Y | NR | NA | Y | 7 |
| 2014 | Chaves et al. | Y | Y | NR | N | Y | NA | NA | Y | Y | N | Y | NR | NA | N | 6 |
| 2014 | Santos et al. | Y | Y | NR | Y | Y | NA | NA | Y | Y | N | Y | NR | NA | N | 7 |
| 2014 | Hayward et al. | Y | Y | Y | Y | N | NA | NA | N | Y | N | Y | NR | NA | N | 6 |
| 2014 | Krause and Hayward | Y | Y | NR | Y | N | NA | NA | N | Y | N | Y | NR | NA | N | 5 |
| 2014 | Lee et al. | Y | Y | NR | Y | N | NA | NA | N | Y | N | Y | NR | NA | N | 5 |
| 2014 | Mefford et al. | Y | Y | NR | N | N | NA | NA | N | Y | N | Y | NR | NA | N | 4 |
| 2014 | Rivera-Ledesma | Y | Y | NR | N | N | NA | NA | N | Y | N | Y | NR | NA | NB | 4 |
| 2013 | Hafeez and Rafique | Y | Y | NR | Y | N | NA | NA | N | Y | N | Y | NR | NA | N | 5 |
| 2013 | Ysseldyk et al. | Y | Y | NR | N | N | NA | NA | N | Y | N | Y | NR | NA | Y | 5 |
| 2012 | Barricelli et al. | Y | Y | Y | Y | N | NA | NA | N | Y | N | Y | NR | NA | N | 6 |
| 2012 | Jahn et al. | Y | Y | NR | N | N | NA | NA | N | Y | N | Y | NR | NA | N | 4 |
| 2012 | Krause and Bastida | Y | Y | NR | Y | N | NA | NA | N | Y | N | Y | NR | NA | Y | 6 |
| 2012 | Momtaz et al. | Y | Y | NR | N | N | NA | NA | Y | Y | N | Y | NR | NA | Y | 6 |
| 2012 | Moon and Kim | Y | Y | Y | N | N | NA | NA | N | Y | N | Y | NR | NA | N | 5 |
| 2012 | Richardson et al. | Y | Y | Y | Y | N | NA | NA | Y | Y | N | Y | NR | NA | Y | 8 |
| 2012 | Vitorino et al. | Y | Y | NR | N | Y | NA | NA | N | Y | N | Y | NR | NA | N | 5 |
| 2012 | Park et al. | Y | Y | Y | Y | N | NA | NA | N | Y | N | Y | NR | NA | Y | 7 |
| 2011 | Callen et al. | Y | Y | Y | NR | N | NA | NA | Y | Y | N | Y | NR | NA | N | 6 |
| 2011 | Correa et al. | Y | Y | NR | Y | Y | NA | NA | N | Y | N | Y | NR | NA | Y | 7 |
| 2011 | Krause et al. | Y | Y | NR | N | N | NA | NA | Y | Y | N | Y | Y | NA | N | 6 |
| 2011 | Lucchetti et al. | Y | Y | Y | Y | N | NA | NA | N | Y | N | Y | NR | NA | Y | 7 |
| 2010 | Schieman and Ellison | Y | Y | Y | Y | N | NA | NA | Y | Y | N | Y | NR | NA | Y | 8 |
| 2009 | Idler et al. | Y | Y | N | Y | N | NA | NA | Y | Y | N | Y | NR | NA | N | 6 |
| 2009 | McFarland | Y | Y | Y | Y | N | NA | NA | Y | Y | N | Y | Y | NA | Y | 9 |
| 2009 | Cardoso e Ferreira | Y | Y | NR | NR | N | NA | NA | N | Y | N | Y | NR | NA | Y | 5 |
| 2009 | Scandrett et al. | Y | Y | NR | N | N | NA | NA | N | Y | N | Y | NR | NA | N | 4 |
| 2009 | Cruz et al. | Y | N | NR | NR | N | NA | NA | N | Y | N | Y | NR | NA | Y | 4 |
| 2008 | Bishop | Y | Y | NR | Y | N | NA | NA | N | Y | N | Y | NR | NA | N | 5 |
| 2008 | Blay et al. | Y | Y | NR | Y | N | NA | NA | N | Y | N | Y | NR | NA | Y | 6 |
| 2008 | Hara et al. | Y | Y | NR | Y | N | NA | NA | N | Y | N | Y | NR | N | N | 5 |
| 2008 | Payman et al. | Y | Y | NR | N | N | NA | NA | Y | Y | N | Y | NR | NA | N | 5 |
| 2008 | Reyes-Ortiz et al. | Y | Y | NR | Y | N | NA | NA | Y | Y | N | Y | NR | NA | N | 6 |
| 2007 | Chaaya et al. | Y | Y | Y | Y | N | NA | NA | Y | Y | N | Y | NR | NA | N | 7 |
| 2007 | Dunn | Y | Y | N | N | N | NA | NA | N | Y | N | Y | NR | NA | N | 4 |
| 2007 | King et al. | Y | Y | Y | N | N | NA | NA | N | Y | N | Y | NR | NA | N | 5 |
| 2007 | Keyes and Reitzes | Y | Y | N | Y | N | NA | NA | N | Y | N | Y | NR | NA | N | 5 |
| 2007 | Yoon and Lee | Y | Y | NR | N | N | NA | NA | N | Y | N | Y | NR | NA | N | 4 |
| 2007 | Chen et al. | Y | Y | NR | Y | N | NA | NA | Y | Y | N | Y | NR | NA | Y | 7 |
| 2006 | Mui and Kang | Y | Y | Y | Y | Y | NA | NA | N | Y | N | Y | NR | NA | N | 7 |
| 2005 | Lee Roff et al. | Y | Y | NR | Y | N | NA | NA | Y | Y | N | Y | NR | NA | N | 6 |
| 2003 | Bosworth et al. | Y | Y | NR | N | N | NA | NA | Y | Y | N | Y | NR | NA | N | 5 |
| 2003 | Meisenhelder | Y | Y | NR | N | N | NA | NA | N | Y | N | Y | NR | NA | N | 4 |
| 2003 | Milstein et al. | Y | Y | NR | Y | Y | NA | NA | N | Y | N | Y | NR | NA | N | 6 |
| 2003 | Parker et al. | Y | Y | NR | Y | N | NA | NA | Y | Y | N | Y | NR | NA | N | 6 |
| 2002 | Blazer et al.* | Y | Y | N | Y | Y | Y | Y | Y | NR | Y | NR | Y | — | — | 9 |
| 2002 | Cicirelli et al. | Y | Y | Y | Y | N | NA | NA | N | Y | N | Y | NR | NA | N | 6 |
| 2002 | Herrera et al. | Y | Y | NR | NR | N | NA | NA | N | N | N | Y | NR | NA | N | 3 |
| 2001 | Braam et al. | Y | Y | NR | NR | NR | NA | NA | N | Y | N | Y | NR | NA | N | 3 |
| 2001 | Fry | Y | Y | NR | Y | N | NA | NA | Y | Y | N | Y | NR | NA | N | 6 |
| 2000 | Musick et al. | Y | Y | Y | Y | Y | NA | NA | Y | Y | N | Y | NR | NA | Y | 9 |
| 2000 | Guglani et al. | Y | Y | NR | N | N | NA | NA | N | Y | N | Y | NR | NA | N | 4 |
| 2000 | Menon et al. | Y | Y | NR | N | N | NA | NA | N | Y | N | Y | NR | NA | Y | 5 |
| 1999 | Husaini et al. | Y | Y | NR | NR | N | NA | NA | N | Y | N | Y | NR | NA | Y | 6 |
| 1998 | Koenig et al. | Y | Y | Y | Y | N | NA | NA | Y | Y | N | Y | NR | NA | Y | 8 |
| 1998 | Musick et al. | Y | Y | Y | Y | Y | NA | NA | Y | Y | N | Y | NR | NA | Y | 9 |
| 1997 | Tapanya et al. | Y | Y | NR | N | N | NA | NA | Y | Y | N | Y | NR | NA | N | 5 |
| 1996 | Kennedy et al. | Y | Y | Y | Y | N | NA | NA | N | Y | N | Y | NR | NA | N | 6 |
| 1995 | Krause | Y | Y | Y | Y | N | NA | NA | Y | Y | N | Y | NR | NA | Y | 8 |
| 1992 | Koenig et al. | Y | Y | NR | Y | N | NA | NA | N | Y | N | Y | NR | NA | N | 5 |
| 1990 | Pressman et al. | Y | Y | Y | Y | N | NA | NA | N | Y | N | Y | NR | NA | N | 6 |
| 1989 | Thorson and Powell | Y | Y | NR | N | N | NA | NA | N | Y | N | Y | NR | NA | N | 4 |
| 1982 | Guy | Y | Y | NR | NR | N | NA | NA | Y | Y | N | Y | NR | NA | N | 5 |
| 1985 | Hunsberger | Y | Y | NR | NR | N | NA | NA | N | Y | N | Y | NR | NA | N | 4 |
| 1977 | Nelson | Y | Y | NR | N | N | NA | NA | N | Y | N | Y | NR | NA | N | 4 |
| 1978 | Reid et al. | Y | Y | NR | Y | N | NA | NA | Y | Y | N | Y | NR | NA | N | 6 |
| *Spirituality* | | | | | | | | | | | | | | | | |
| 2021 | Khodarahimi et al. | Y | Y | NR | N | N | NA | NA | N | Y | N | Y | NR | NA | N | 4 |
| 2020 | Aydin et al. | Y | Y | NR | N | Y | NA | NA | N | Y | N | Y | NR | NA | N | 5 |
| 2020 | Fernandes et al. | Y | Y | NR | N | N | NA | NA | N | Y | N | Y | NR | NA | N | 4 |
| 2020 | Ilyas et al. | Y | Y | NR | N | N | NA | NA | N | Y | N | Y | NR | NA | Y | 5 |
| 2019 | Hassoun et al. | Y | Y | N | N | N | NA | NA | Y | Y | N | Y | NR | NA | N | 5 |
| 2019 | Salman and Lee | Y | Y | Y | Y | Y | NA | NA | N | Y | N | Y | NR | NA | N | 7 |
| 2018 | Thauvoye et al. | Y | Y | Y | Y | N | NA | NA | N | Y | N | Y | NR | NA | N | 6 |
| 2017 | Araújo et al. | Y | Y | Y | Y | N | NA | NA | N | Y | N | Y | NR | NA | N | 6 |
| 2017 | Garces et al. | Y | Y | NR | Y | N | NA | NA | N | Y | N | Y | NR | NA | N | 5 |
| 2017 | Lee and Salman | Y | Y | N | Y | Y | NA | NA | N | Y | N | Y | NR | NA | Y | 7 |
| 2017 | Pilger et al. | Y | Y | Y | N | N | NA | NA | N | Y | N | Y | NR | NA | N | 5 |
| 2017 | Souza et al. | Y | Y | Y | Y | N | NA | NA | Y | Y | N | Y | NR | NA | N | 7 |
| 2016 | Vitorino et al. | Y | Y | NR | Y | Y | NA | NA | Y | Y | N | Y | NR | NA | N | 7 |
| 2015 | Ali et al. | Y | Y | NR | Y | N | NA | NA | N | Y | N | Y | NR | NA | N | 5 |
| 2015 | Jun e Bolin | Y | Y | NR | Y | N | NA | NA | Y | Y | N | Y | NR | NA | N | 6 |
| 2015 | Oliver et al. | Y | Y | NR | N | N | NA | NA | N | Y | N | Y | NR | NA | Y | 5 |
| 2014 | Caldeira et al. | Y | Y | NR | N | N | NA | NA | N | Y | N | Y | NR | NA | N | 4 |
| 2012 | Park and Roh | Y | Y | NR | N | N | NA | NA | N | Y | N | Y | NR | NA | Y | 5 |
| 2011 | Vahia et al. | Y | Y | Y | Y | N | NA | NA | N | Y | N | Y | NR | NA | N | 6 |
| 2011 | Coleman et al. | Y | Y | NR | N | N | NA | NA | N | Y | N | Y | NR | NA | N | 4 |
| 2011 | Lee and Yoon | Y | Y | NR | Y | N | NA | NA | N | Y | N | Y | NR | NA | N | 5 |
| 2010 | Skarupski et al. | Y | Y | NR | Y | N | NA | NA | N | Y | N | Y | NR | NA | N | 5 |
| 2009 | You et al. | Y | Y | NR | N | N | NA | NA | N | Y | N | Y | NR | NA | Y | 5 |
| 2004 | Kirby et al. | Y | Y | NR | N | N | NA | NA | N | Y | N | Y | NR | NA | Y | 5 |
| 2000 | Meisenhelder and Chandler | Y | Y | N | Y | N | NA | NA | N | Y | N | Y | NR | NA | N | 5 |
| Y= Yes; N= No; NA= Not applide; NR= Not reported; P= Probably; *Case-control | | | | | | | | | | | | | | | | |
