# Supplementary material for: Religiosity/Spirituality and Mental Health in Older Adults: A Systematic Review and Meta-Analysis of Observational Studies
Source: Front Med (Lausanne). 2022 May 12;9:877213. doi: 10.3389/fmed.2022.877213 (PMC9133607; doi:10.3389/fmed.2022.877213)
Supplement: Supplementary file 5 [file Data_Sheet_5.docx]

| **Supplementary Material 5**. Quality analysis | | | | | | | | | | | | | | | | |
| --- | --- | --- | --- | --- | --- | --- | --- | --- | --- | --- | --- | --- | --- | --- | --- | --- |
| **Year** | **Authors** | **1** | **2** | **3** | **4** | **5** | **6** | **7** | **8** | **9** | **10** | **11** | **12** | **13** | **14** | **Overall score (0/14)** |
| *Longitudinal* | | | | | | | | | | | | | | | | |
| Year | Author |  |  |  |  |  |  |  |  |  |  |  |  |  |  |  |
| 2018 | Jung | Y | Y | Y | Y | N | Y | Y | N | Y | N | Y | NR | N | Y | 9 |
| 2015 | Roh et al. | Y | Y | Y | Y | N | Y | Y | Y | Y | N | Y | NR | N | Y | 10 |
| 2014 | Hui-Chuan | Y | Y | NR | Y | N | Y | Y | Y | Y | N | Y | NR | NR | N | 8 |
| 2013 | Ysseldyk et al. | Y | Y | NR | N | N | Y | Y | N | Y | N | Y | NR | N | Y | 7 |
| 2012 | Sun et al. | Y | Y | Y | Y | N | Y | Y | Y | Y | N | Y | NR | N | N | 9 |
| 2009 | Law and Sbarra | Y | Y | NR | Y | N | Y | Y | Y | Y | N | Y | NR | NR | N | 8 |
| 2008 | Norton et al. | Y | Y | Y | Y | N | Y | Y | Y | Y | N | Y | NR | Y | Y | 11 |
| 1996 | Kivela et al. | Y | Y | Y | Y | N | Y | Y | N | Y | N | Y | NR | N | N | 8 |
| Y= Yes; N= No; NA= Not applide; NR= Not reported; P= Probably; *Case-control | | | | | | | | | | | | | | | | |
