# Supplementary material for: Religiosity/Spirituality and Mental Health in Older Adults: A Systematic Review and Meta-Analysis of Observational Studies
Source: Front Med (Lausanne). 2022 May 12;9:877213. doi: 10.3389/fmed.2022.877213 (PMC9133607; doi:10.3389/fmed.2022.877213)
Supplement: Supplementary file 6 [file Data_Sheet_6.docx]

| **Supplementary Material 6**. Quality Assessment | | | | | | | | | | | |
| --- | --- | --- | --- | --- | --- | --- | --- | --- | --- | --- | --- |
| *Religiosity, Cross-Sectional* | | | | | | | | |  |  | |
| **Year** | **Authors** | 1 | 2 | 3 | 4 | 1 | 1 | 2 |  | Quantitative Score | Risk of bias |
| 2020 | Aslan et al. | d | b | a | a | — | c | b |  | 4 | High |
| 2020 | Foong et al. | a | b | a | a | a,b | c | a |  | 8 | Low |
| 2020 | Bae | a | b | a | a | — | c | b |  | 5 | Moderate |
| 2020 | Abdel-Hady & El-Gilany | a | a | a | a | — | c | b |  | 6 | Moderate |
| 2020 | Mitchell et al. | a | b | a | a | a,b | c | a |  | 8 | Low |
| 2020 | Molina et al. | b | a | c | a | — | c | b |  | 5 | Moderate |
| 2020 | Sharif et al. | b | a | b | a | a,b | c | a |  | 8 | Low |
| 2020 | Solaimanizadeh et al. | d | b | c | a | — | c | b |  | 3 | High |
| 2020 | Gallardo-Peralta and Sánchez-Moreno | b | b | c | a | a,b | c | a |  | 7 | Low |
| 2019 | Silva et al. | c | b | c | a | — | c | b |  | 3 | High |
| 2019 | Bakhtiari et al. | b | b | c | a | — | c | b |  | 4 | High |
| 2019 | Bakan et al. | c | b | b | a | a,b | c | a |  | 6 | Moderate |
| 2019 | Fernández-Niño et al. | a | b | a | a | a,b | c | a |  | 8 | Low |
| 2019 | Hamid et al. | a | b | a | a | a,b | c | a |  | 8 | Low |
| 2019 | Hill et al. | a | b | a | a | a,b | c | a |  | 8 | Low |
| 2019 | Reyes-Ortiz et al. | d | a | a | a | — | c | b |  | 5 | Moderate |
| 2019 | Strinnholm et al. | b | b | a | a | — | c | b |  | 5 | Moderate |
| 2019 | Willis et al. | b | b | c | a | — | c | b |  | 4 | High |
| 2019 | Moreno et al. | d | b | c | a | — | c | b |  | 3 | High |
| 2019 | Ejiri et al. | a | b | a | a | a,b | c | a |  | 8 | Low |
| 2018 | El-Gilany et al. | b | a | a | a | — | c | b |  | 6 | Moderate |
| 2018 | Kotian et al. | b | b | b | a | — | c | b |  | 4 | High |
| 2018 | Manning and Miles | a | a | c | a | — | c | b |  | 5 | Moderate |
| 2018 | Nery et al. | c | b | b | a | — | c | b |  | 3 | High |
| 2018 | Munawar and Tariq | b | b | c | a | — | c | b |  | 4 | High |
| 2017 | Jung et al. | c | b | b | a | — | c | a |  | 3 | High |
| 2017 | Lac et al. | c | b | c | a | — | c | b |  | 3 | High |
| 2017 | Nunes et al. | c | b | b | a | — | c | b |  | 3 | High |
| 2016 | Bonnewyn et al. | c | b | a | a | a,b | c | a |  | 7 | Low |
| 2016 | McGowan et al. | c | b | c | a | a,b | c | a |  | 6 | Moderate |
| 2016 | Krok | a | b | c | a | — | c | b |  | 4 | High |
| 2016 | Vieira and Aquino | c | b | c | a | — | c | b |  | 3 | High |
| 2015 | Abdala et al. | c | b | c | a | a,b | c | a |  | 6 | Moderate |
| 2015 | Fastame et al. | c | b | a | a | — | c | b |  | 4 | High |
| 2015 | Stecz and Kocur | c | b | a | a | — | c | b |  | 4 | High |
| 2014 | Andrade | a | a | c | a | — | c | b |  | 5 | Moderate |
| 2014 | Feng et al. | a | b | a | a | a,b | c | a |  | 8 | Low |
| 2014 | Chaves et al. | a | a | c | a | — | c | b |  | 5 | Moderate |
| 2014 | Santos et al. | a | a | c | a | — | c | b |  | 5 | Moderate |
| 2014 | Hayward et al. | a | b | a | a | — | c | b |  | 5 | Moderate |
| 2014 | Krause and Hayward | a | b | c | a | — | c | b |  | 4 | High |
| 2014 | Lee et al. | b | b | c | a | — | c | b |  | 4 | High |
| 2014 | Mefford et al. | c | b | c | a | — | c | b |  | 3 | High |
| 2014 | Rivera-Ledesma | c | b | c | a | — | c | b |  | 3 | High |
| 2013 | Hafeez and Rafique | b | b | c | a | — | c | b |  | 4 | High |
| 2012 | Barricelli et al. | c | b | a | a | — | c | b |  | 4 | High |
| 2012 | Jahn et al. | c | b | c | a | — | c | b |  | 3 | High |
| 2012 | Krause and Bastida | a | b | c | a | a,b | c | a |  | 7 | Low |
| 2012 | Momtaz et al. | b | b | c | a | a,b | c | a |  | 7 | Low |
| 2012 | Moon and Kim | c | b | a | a | — | c | b |  | 4 | High |
| 2012 | Richardson et al. | b | b | a | a | a,b | c | a |  | 8 | Low |
| 2012 | Vitorino et al. | c | a | c | a | — | c | b |  | 4 | High |
| 2012 | Park et al. | d | b | a | a | a,b | c | a |  | 7 | Low |
| 2011 | Callen et al. | d | b | a | a | — | c | b |  | 4 | High |
| 2011 | Correa et al. | b | a | c | a | a,b | c | a |  | 8 | Low |
| 2011 | Krause et al. | b | b | c | a | — | c | b |  | 4 | High |
| 2011 | Lucchetti et al. | c | b | a | a | a,b | c | a |  | 7 | Low |
| 2010 | Schieman and Ellison | a | b | a | a | a,b | c | a |  | 8 | Low |
| 2009 | Idler et al. | b | b | b | a | — | c | b |  | 4 | High |
| 2009 | McFarland | a | b | a | a | a,b | c | a |  | 8 | Low |
| 2009 | Cardoso e Ferreira | d | b | c | a | a,b | c | a |  | 6 | Moderate |
| 2009 | Scandrett et al. | c | b | c | a | — | c | b |  | 3 | High |
| 2009 | Cruz et al. | d | b | c | a | a,b | c | a |  | 6 | Moderate |
| 2008 | Bishop | c | b | c | a | — | c | b |  | 3 | High |
| 2008 | Blay et al. | a | b | c | a | a,b | c | a |  | 7 | Low |
| 2008 | Hara et al. | b | b | c | a | — | c | b |  | 4 | High |
| 2008 | Payman et al. | c | b | c | a | — | c | b |  | 3 | High |
| 2008 | Reyes-Ortiz et al. | b | b | c | a | — | c | b |  | 4 | High |
| 2007 | Chaaya et al. | a | b | a | a | — | c | b |  | 5 | Moderate |
| 2007 | Dunn | a | b | b | a | — | c | b |  | 4 | High |
| 2007 | King et al. | b | b | a | a | — | c | b |  | 5 | Moderate |
| 2007 | Keyes and Reitzes | b | b | b | a | — | c | b |  | 4 | High |
| 2007 | Yoon and Lee | c | b | c | a | — | c | b |  | 3 | High |
| 2007 | Chen et al. | b | b | c | a | a,b | c | a |  | 7 | Low |
| 2006 | Mui and Kang | a | a | a | a | — | c | b |  | 6 | Moderate |
| 2005 | Lee Roff et al. | a | b | c | a | — | c | b |  | 4 | High |
| 2003 | Bosworth et al. | c | b | c | a | — | c | b |  | 3 | High |
| 2003 | Meisenhelder | a | b | c | a | — | c | b |  | 4 | High |
| 2003 | Milstein et al. | a | a | c | a | — | c | b |  | 5 | Moderate |
| 2003 | Parker et al. | a | b | c | a | — | c | b |  | 4 | High |
| 2002 | Cicirelli et al. | b | b | a | a | — | c | b |  | 5 | Moderate |
| 2002 | Herrera et al. | c | b | c | a | — | c | b |  | 3 | High |
| 2001 | Braam et al. | b | b | c | a | — | c | b |  | 4 | High |
| 2001 | Fry | c | b | c | a | — | c | b |  | 3 | High |
| 2000 | Musick et al. | a | a | a | a | a,b | c | a |  | 9 | Low |
| 2000 | Guglani et al. | b | b | c | a | — | c | b |  | 4 | High |
| 2000 | Menon et al. | c | b | c | a | a,b | c | a |  | 6 | Moderate |
| 1999 | Husaini et al. | d | b | c | a | a,b | c | a |  | 6 | Moderate |
| 1998 | Koenig et al. | a | b | a | a | a,b | c | a |  | 8 | Low |
| 1998 | Musick et al. | a | a | a | a | a,b | c | a |  | 9 | Low |
| 1997 | Tapanya et al. | d | b | c | a | — | c | b |  | 3 | High |
| 1996 | Kennedy et al. | a | b | a | a | — | c | b |  | 5 | Moderate |
| 1995 | Krause | a | b | a | a | a,b | c | a |  | 8 | Low |
| 1992 | Koenig et al. | a | b | c | a | — | c | b |  | 4 | High |
| 1990 | Pressman et al. | c | b | a | a | — | c | b |  | 4 | High |
| 1989 | Thorson and Powell | c | b | c | a | — | c | b |  | 3 | High |
| 1982 | Guy | b | b | c | a | — | c | b |  | 4 | High |
| 1985 | Hunsberger | c | b | c | a | — | c | b |  | 3 | High |
| 1977 | Nelson | c | b | c | a | — | c | b |  | 3 | High |
| 1978 | Reid et al. | a | b | c | a | — | c | b |  | 4 | High |
|  |  |  |  |  |  |  |  |  |  |  |  |
| *Religiosity, Case-Control* | | | | | | | | |  |  | |
| **Year** | **Authors** | 1 | 2 | 3 | 4 | 1 | 1 | 2 |  | Quantitative Score | Risk of bias |
| 2002 | Blazer et al.* | b | a | a | a | — | c | a |  | 5 | Moderate |
|  |  |  |  |  |  |  |  |  |  |  |  |
| *Spirituality, Cross-Sectional* | | | | | | | | |  |  | |
| **Year** | **Authors** | 1 | 2 | 3 | 4 | 1a | 1 | 2 |  |  |  |
| 2021 | Khodarahimi et al. | c | b | c | a | — | c | b |  | 3 | High |
| 2020 | Aydin et al. | c | a | c | a | — | c | b |  | 4 | High |
| 2020 | Fernandes et al. | d | b | c | a | — | c | b |  | 3 | High |
| 2020 | Ilyas et al. | b | b | c | a | a,b | c | a |  | 7 | Low |
| 2020 | Gallardo-Peralta and Sánchez-Moreno | b | b | c | a | a,b | c | a |  | 7 | Low |
| 2019 | Hassoun et al. | b | b | b | a | — | c | b |  | 4 | High |
| 2019 | Salman and Lee | b | a | a | a | — | c | b |  | 6 | Moderate |
| 2019 | Moreno et al. | d | b | c | a | — | c | b |  | 3 | High |
| 2018 | Thauvoye et al. | c | b | a | a | — | c | b |  | 4 | High |
| 2017 | Araújo et al. | c | b | a | a | — | c | b |  | 4 | High |
| 2017 | Garces et al. | c | b | c | a | — | c | b |  | 3 | High |
| 2017 | Lee and Salman | b | a | b | a | a,b | c | a |  | 8 | Low |
| 2017 | Pilger et al. | c | b | a | a | — | c | b |  | 4 | High |
| 2017 | Souza et al. | b | b | a | a | — | c | b |  | 5 | Moderate |
| 2016 | Vitorino et al. | b | a | c | a | — | c | b |  | 5 | Moderate |
| 2015 | Ali et al. | a | b | c | a | — | c | b |  | 4 | High |
| 2015 | Jun e Bolin | b | b | c | a | — | c | b |  | 4 | High |
| 2015 | Oliver et al. | c | b | c | a | a,b | c | a |  | 6 | Moderate |
| 2014 | Caldeira et al. | d | b | c | a | — | c | b |  | 3 | High |
| 2012 | Park and Roh | c | b | c | a | a,b | c | a |  | 6 | Moderate |
| 2011 | Vahia et al. | b | b | a | a | — | c | b |  | 5 | Moderate |
| 2011 | Coleman et al. | c | b | c | a | — | c | b |  | 3 | High |
| 2011 | Lee and Yoon | b | b | c | a | — | c | b |  | 4 | High |
| 2010 | Skarupski et al. | a | b | c | a | — | c | b |  | 4 | High |
| 2009 | You et al. | c | b | c | a | a,b | c | a |  | 6 | Moderate |
| 2004 | Kirby et al. | b | b | c | a | a,b | c | a |  | 7 | Low |
| 2000 | Meisenhelder and Chandler | a | b | b | a | — | c | b |  | 4 | High |
|  |  |  |  |  |  |  |  |  |  |  |  |
| *Religiosity, Longitudinal* | | | | | | | | |  |  | |
| 2018 | Jung | b | a | b | a | b | c | a | c | 7 | Good |
| 2015 | Roh et al. | a | a | b | a | b | c | a | c | 6 | Good |
| 2014 | Hui-Chuan | a | a | b | a | c | c | a | d | 5 | Good |
| 2013 | Ysseldyk et al. | b | a | b | a | b | c | a | c | 6 | Good |
| 2012 | Sun et al. | b | a | b | a | c | c | a | c | 5 | Good |
| 2009 | Law and Sbarra | a | a | b | a | c | c | a | d | 5 | Good |
| 2008 | Norton et al. | b | a | b | a | b | a | a | b | 8 | Good |
| 1996 | Kivela et al. | b | a | b | a | c | a | a | c | 6 | Good |
